# Exome sequencing in 38 patients with intracranial aneurysms and subarachnoid hemorrhage

**DOI:** 10.1007/s00415-020-09865-6

**Published:** 2020-05-04

**Authors:** Thomas Sauvigny, Malik Alawi, Linda Krause, Sina Renner, Michael Spohn, Alice Busch, Verena Kolbe, Janine Altmüller, Britt-Sabina Löscher, Andre Franke, Christian Brockmann, Wolfgang Lieb, Manfred Westphal, Nils Ole Schmidt, Jan Regelsberger, Georg Rosenberger

**Affiliations:** 1 Department of Neurosurgery, University Medical Center Hamburg-Eppendorf, Martinistraße 52, 20246 Hamburg, Germany; 2 Bioinformatics Core, University Medical Center Hamburg-Eppendorf, Martinistraße 52, 20246 Hamburg, Germany; 3 Institute of Medical Biometry and Epidemiology, University Medical Center Hamburg-Eppendorf, Martinistraße 52, 20246 Hamburg, Germany; 4 Institute of Human Genetics, University Medical Center Hamburg-Eppendorf, Martinistraße 52, 20246 Hamburg, Germany; 5 Research Institute Children’s Cancer Center Hamburg, Martinistraße 52, 20251 Hamburg, Germany; 6 Department of Oncology, Hematology and Bone Marrow Transplantation With Section Pneumology, Hubertus Wald Tumorzentrum, University Comprehensive Cancer Center Hamburg, University Medical Center Hamburg-Eppendorf, Martinistraße 52, 20246 Hamburg, Germany; 7 Cologne Center for Genomics, Center for Molecular Medicine Cologne (CMMC), University of Cologne, Weyertal 115b, 50931 Cologne, Germany; 8 Institute of Clinical Molecular Biology, Christian-Albrechts-University Kiel, Rosalind-Franklin-Straße 12, 24105 Kiel, Germany; 9 Institute of Transfusion Medicine, University Hospital of Schleswig-Holstein, Campus Lübeck, Ratzeburger Allee 160, 23538 Lübeck, Germany; 10 Institute of Epidemiology, Christian-Albrechts-University Kiel, Niemannsweg 11, 24105 Kiel, Germany; 11 Department of Neurosurgery, University Medical Center Regensburg, Franz-Josef-Strauss-Allee 11, 93053 Regensburg, Germany

**Keywords:** Subarachnoid hemorrhage, Intracranial aneurysms, Disease gene identification, Exome sequencing, *EDIL3*

## Abstract

**Objective:**

Genetic risk factors for unruptured intracranial aneurysms (UIA) and aneurysmal subarachnoid hemorrhage (aSAH) are poorly understood. We aimed to verify recently reported risk genes and to identify novel sequence variants involved in the etiology of UIA/aSAH.

**Methods:**

We performed exome sequencing (ES) in 35 unrelated individuals and 3 family members, each with a history of UIA and/or aSAH. We searched for sequence variants with minor allele frequency (MAF) ≤ 5% in the reported risk genes *ADAMTS15*, *ANGPTL6*, *ARHGEF17*, *LOXL2*, *PCNT*, *RNF213*, *THSD1* and *TMEM132B*. To identify novel putative risk genes we looked for unknown (MAF = 0) variants shared by the three relatives.

**Results:**

We identified 20 variants with MAF ≤ 5% in 18 individuals: 9 variants in *PCNT* (9 patients), 4 in *RNF213* (3 patients), 3 in *THSD1* (6 patients), 2 in *ANGPTL6* (3 patients), 1 in *ADAMTS15* (1 patient) and 1 in *TMEM132B* (1 patient). In the affected family, prioritization of shared sequence variants yielded five novel putative risk genes. Based on predicted pathogenicity of identified variants, population genetics data and a high functional relevance for vascular biology, *EDIL3* was selected as top candidate and screened in additional 37 individuals with UIA and/or aSAH: a further very rare *EDIL3* sequence variant in two unrelated sporadic patients was identified.

**Conclusions:**

Our data support a role of sequence variants in *PCNT*, *RNF213* and *THSD1* as susceptibility factors for cerebrovascular disease. The documented function in vascular wall integrity, the crucial localization of affected amino acids and gene/variant association tests suggest *EDIL3* as a further valid candidate disease gene for UIA/aSAH.

**Electronic supplementary material:**

The online version of this article (10.1007/s00415-020-09865-6) contains supplementary material, which is available to authorized users.

## Introduction

Aneurysmal subarachnoid hemorrhage (aSAH) affects approximately 500,000 individuals per year worldwide and, despite immense medical attempts, is associated with high mortality [1]. The risk/prevalence of intracranial aneurysms (IA) in the general population is even higher and estimated at around 3% [2]. Besides life-style and medical risk factors (e.g. smoking, hypertension), the risk for IA and aSAH is increased among patient’s family members. However, the genetic background of the disease is still poorly understood. Although large genome or exome wide association studies (GWAS or EWAS) identified several risk loci, valid disease genes have not been established [3]. In addition to GWAS providing associations between genomic regions and a disease, novel advanced approaches using exome sequencing (ES) promise to detect causal relations. Indeed, recent studies applied ES in families affected with IA/SAH and identified alterations in *ANGPTL6* (angiopoietin-like 6), *RNF213* (ring finger protein 213), *THSD1* (thrombospondin type 1 domain containing protein 1), *ARHGEF17* (Rho guanine nucleotide exchange factor 17) and *PCNT* (pericentrin) as genetic risk factors for this disease [4–8]. Other family-based ES studies reported on various candidate disease variants and genes including *ADAMTS15*, *LOXL2* and *TMEM132B*, and emphasize the need of validation of their presented data [9–11]. The a.m. studies on genetic risk factors for IA/SAH differ in the resulting strength of evidence (for details see Supplementary Introduction).

Clearly, the significance of ES data highly depends on thorough clinical phenotypic characterization because, in addition to heritable conditions, IA/SAH is also associated with acquired risk factors [12]. Since a genetic background may well be of importance and significance for both, unruptured intracranial aneurysms (UIA) and aSAH [13, 14], we established and describe here a cohort comprising individuals with a familial history of UIA and/or aSAH and substantially affected sporadic patients with UIA and/or aSAH. We performed ES in 38 patients to validate already reported candidate genes for IA/SAH on the one hand, and to identify novel susceptibility genes on the other. We completed our genetic screen by sequencing the top candidate gene in the entire patient cohort.

## Methods

### Study cohort

Individuals of Northwestern German descent (genetically belonging to the group of non-Finnish, Northwestern Europeans) were recruited in the Interdisciplinary Neurovascular Center of the University Medical Center Hamburg-Eppendorf, Hamburg, Germany; approximately 70 patients with aSAH and 200 patients with intracranial aneurysms in total are treated there per year. Inclusion–exclusion criteria were as follows: (i) Only individuals with a family history of UIA and/or aSAH and substantially affected sporadic patients with ≥ 2 UIA or ≥ 1 UIA and aSAH were included. To that, we selected patients based on age, number of aneurysms and family history, resulting in a collective of relatively young patients with an increased number of aneurysms and family history above average compared to data provided in the literature (Table 1) [15, 16]. Experienced physicians assessed the patients; familial history, clinical appearance, primary risk conditions (e.g. hypertension) and life-style risk factors (e.g. smoking) were recorded (Table 1). Missing clinical data of patients previously treated in a different institution were gathered as far as possible. Phenotype definitions following the FIA study protocol [9]: “UIA and/or aSAH” (individuals having a documented UIA based on intra-arterial angiogram or operative report; individuals having an aSAH documented in medical records based on CT/MRI-scan, intra-arterial angiogram or operative report); “probable aSAH” (aSAH was stated in the medical history with supporting documentation such as severe headache or altered level of consciousness). (ii) Informed consents for blood collection, DNA storage and genetic analyses were obtained from all study participants. (iii) Patients with a familial or personal history of disorders associated with IA (i.e. polycystic kidney disease, Ehlers–Danlos syndrome, Loeys–Dietz syndrome and Marfan syndrome) were excluded based on clinical findings. In total, we screened 101 individuals, of which 75 met the described inclusion criteria. To assess the putative risk of rupture in patients with UIA in comparison to those with a proven aneurysmal bleeding, we calculated the PHASES score (Table 1) [17]. In patients with multiple UIAs, the score was calculated for each aneurysm site and size, and the highest score was documented (Table 1). In the aSAH group, the score for the ruptured aneurysm was calculated retrospectively (Table 1). Finally, we assessed clinical characteristics for following subgroups (Table 1): exome sequenced individuals (ES IND), individuals with positive family history (FAM IND) and substantially affected sporadic individuals (SPO IND).

**Table 1:** Clinical characteristics

Characteristics	All (n = 75) Mean ± SD (range)	aSAH (*N* = 48) Mean ± SD (range)	UIA (*N* = 27) Mean ± SD (range)	ES IND (*n* = 35) Mean ± SD (range)	FAM IND (*n* = 30) Mean ± SD (range)	SPO IND (*N* = 45) Mean ± SD (range)
Age at SAH (years)		46.9 ± 12.1 (9–71)				
IA (count)	1.96 ± 1.40 (1–6)	1.8 ± 1.3 (0–6)	2.3 ± 1.6 (1–6)	2.23 ± 1.57 (1–6)	1.80 ± 1.27 (1–6)	2.07 ± 1.48 (1–6)
Aneurysm diameter (mm)	7.1 ± 6.6 (1.5–47.0)	5.7 ± 2.6 (1.5–13.0)	9.6 ± 910.2 (2.0–47.0)	5.76 ± 3.22 (1.5–47.0)	6.75 ± 5.95 (2.0–30.0)	7.35 ± 7.07 (1.5–47.0)
Phases score	4.8 (0–13)	4.7 (0–12)	5.0 (0–13)	4.8 (0–13)	4.5 (0–13)	5.0 (0–12)
Hunt and hess grade		2.3 (1–5)				
WFNS grade		1.9 (1–5)				
Fisher score		3.4 (1–4)				
Outcome at discharge (EGOS)		4.9 (1–8)				
LAO (months)		83 ± 298 (2–1343)				
Outcome LAO (MRS)		1.3 (0–6)				

	**Count (%)**	**Count (%)**	**Count (%)**	**Count (%)**	**Count (%)**	**Count (%)**
**Sex**						
Female	53 (70.7)	35 (72.9)	18 (66.7)	26 (74.3)	22 (73.3)	31 (68.9)
Male	22 (29.3)	13 (17.3)	9 (33.3)	9 (25.7)	8 (26.7)	14 (31.1)
**Aneurysm location**^a^						
Anterior circulation	56 (80.0)	34 (77.3)	22 (84.6)	25 (75.8)	22 (81.5)	34 (79.1)
Posterior circulation	11 (15.7)	10 (22.7)	1 (3.8)	7 (21.2)	5 (18.5)	6 (14.0)
Both	3 (4.3)	–	3 (11.5)	1 (3.0)	–	3 (7.0)
Unknown	5			2	3	2
**Family history**						
Yes	30 (40)	13 (27.1)	17 (63.0)	14 (60.0)	30 (100.0)	0 (0.0)
No	45 (60)	35 (72.9)	10 (37.0)	21 (40.0)	0 (0.0)	45 (100.0)
**Vascular risk factors**						
Hypertension	27 (36.5)	13 (27.7)	14 (51.9)	13 (37.1)	12 (41.4)	15 (33.3)
Diabetes	3 (4.1)	1 (2.1)	2 (7.4)	1 (2.9)	1 (3.4)	2 (4.4.)
Hyperlipidemia	6 (8.1)	3 (6.4)	3 (11.1)	4 (11.4)	3 (10.3)	3 (6.7)
Prev. cardiovas. disease	4 (5.5)	2 (4.3)	2 (7.4)	2 (5.7)	1 (3.4)	3 (6.8)
Prev. neurol. disease	12 (16.4)	9 (19.6)	3 (11.1)	9 (25.7)	1 (3.4)	7 (15.9)
Ever smoker	41 (56.2)	28 (59.6)	13 (50.0)	20 (57.1)	18 (62.1)	23 (52.3)
Alcohol	2 (2.7)	2 (4.3)	0 (0.0)	1 (2.9)	0 (0.0)	2 (4.4)
**Treatment**						
Endovascular	34 (47.2)	29 (63.0)	5 (47.2)	12 (36.4)	8 (29.6)	26 (57.8)
Surgical	27 (37.5)	13 (28.3)	14 (53.8)	15 (45.5)	12 (44.4)	15 (33.3)
Both	11 (15.3)	4 (8.7)	7 (26.9)	6 (18.2)	7 (25.9)	4 (8.9)
No	–	–	–	–	–	–
Unknown	3			2	3	
**DCI**						
Yes		16 (38.1)				
No		26 (61.9)				
Unknown		6				

*SD* standard deviation, *aSAH* aneurysmal subarachnoid hemorrhage, *UIA* unruptured intracranial aneurysm, *ES IND* exome sequenced individuals, *FAM IND* individuals with positive family history, *SPO IND* substantially affected sporadic individuals, *IA* intracranial aneurysm, *BMI* body mass index, *GCS* Glasgow Coma Scale, *WFNS* World Federation of Neurosurgical Societies, *EGOS* Extended Glasgow Outcome Scale, *LAO* last available outcome, *MRS* Modified Rankin Scale, *DCI* delayed cerebral injury

^a^Only the ruptured aneurysm was reported in the aSAH group

### Exome sequencing, variant calling, filtering strategies, variant prioritization and pathogenicity assignment

Exome sequencing (ES) is a genomic technique for sequencing all coding regions (= exome) of a genome: after capturing only the subset of DNA that encodes proteins, the exome is sequenced using a high-throughput DNA sequencing technology. We performed ES in 35 unrelated individuals and 3 affected members of a family. Selection criteria and constraints of subjects for exome and Sanger sequencing are explained in detail in Supplementary Methods and Table S1. Although genome-wide association studies (GWAS) that focus on the identification of common variants (minor allele frequency, MAF > 5%) revealed a large number of risk loci associated with IA/SAH, the accounted heritability in these loci was low [18]. The prevalence of IA is reported to be approximately 5% in average [2]; for genetic disorders the phenotype prevalence and penetrance should be used to define the allele frequency cutoff [19]. Therefore, for the validation of reported UIA/SAH risk genes, we screened for low-frequency (MAF > 1 to 5%), rare (MAF > 0.1 to 1%), very rare (MAF ≤ 0.1%) and unknown variants (MAF = 0) in these genes [20, 21], thereby including those variants with strong, intermediate as well as moderate effect size and penetrance [22]. For the identification of novel susceptibility genes in the family-based approach we searched for unknown variants (MAF = 0; with anticipated significant effect size/penetrance) shared by the affected family members. Subsequent variant prioritization, the top familial candidate disease gene was examined in the remaining 37 patients from our cohort.

Detailed information on exome sequencing, variant calling, filtering strategy, variant prioritization, pathogenicity assignment, variant classification, mutation analysis, presentation of clinical data, statistical analysis (gene association tests), molecular modeling as well as databases and repositories is outlined in Supplementary Methods.

## Results

### Clinical characteristics

Our study cohort comprises 75 subjects (53 female; 70.7%) including 48 individuals (64.0%) with aneurysmal subarachnoid bleeding (aSAH group, Table 1) and 27 patients (36.0%) with UIA (UIA group, Table 1). 31 patients (41.3%) showed multiple IA (including ruptured and unruptured), the mean count of aneurysms was 2.3 in the UIA group and 1.8 in the aSAH group. In the latter group, 5 patients (10.4%) suffered from a previous aSAH. In addition to UIA and/or aSAH, three patients (4.0%) had an aortic aneurysm. 30 individuals (40.0%) reported a family history of IA and/or SAH. The mean PHASES score was 4.8. Clinical data are summarized in Table 1. Taken together, this cohort consists of individuals with a family history of UIA and/or aSAH and substantially affected patients with UIA and/or aSAH.

### Genetic analysis of reported risk genes

38 patients underwent ES. The presence of possibly pathogenic variants in known disease genes for vascular/connective tissue disorders was excluded (details are given in Supplementary Methods and Table S2). Subsequently, exome data were analyzed for variants in the reported risk genes *ADAMTS15*, *ANGPTL6*, *ARHGEF17*, *LOXL2*, *PCNT*, *RNF213*, *THSD1* and *TMEM132B*. In total we found 20, exclusively heterozygous missense variants with MAF ≤ 0.05 (5%) in 6 genes (Table 2): 9 *PCNT* variants were identified in 9 patients, 4 *RNF213* variants in 3 patients, 3 *THSD1* variants in 6 patients, 2 *ANGPTL6* variants in 3 patients, and 1 variant in *ADAMTS15* and *TMEM132B* in 1 patient each. No variants were detected in *ARHGEF17* and *LOXL2*. According to ACMG/AMP guidelines [23], we defined 17 variants of uncertain significance (VUS) and 3 likely benign variants (LBV) (Table 2). Details on criteria for classifying variants are given in Table 2 and in Supplementary Methods. All detected variants were annotated in dbSNP (www.ncbi.nlm.nih.gov/SNP/) and gnomAD [24]; we did not find undescribed variants in the reported risk genes.

**Table 2:** Variants in reported risk genes identified in 38 patients with UIA and/or aSAH

Gene gnomAD constraint metrics	Transcript ID	c. notation p. notation	dbSNP rs ID ClinVar allele ID	Classification^a^	gnomAD all MAF^b^ het/hom/Σ	Pathogenicity predictions^c^		Subjects	Clinical findings; family history (FH)
						CADD/REVEL/M-CAP/ClinPred	Impact on splicing		
*ADAMTS15* misZ = 0.60 pLI = 0.0	NM_139055	c.1258G>A p.Gly420Ser	rs767345140 n.r	VUS_PM1+PP3_	0.006092% 17/0/279072	33/0.506/0.011/0.641	last nucleotide in exon 3; donor loss (HSF, NG2, MES)	IA83	1 IA + 1 aSAH FH neg
*ANGPTL6* misZ = 0.64 pLI = 0.0	NM_031917	c.1072G>A p.Arg358Cys	rs147149731 n.r	VUS_PM1+BP4_	1.139% 3221/32/282740	13.39/0.238/n.a./0.014	n.d	IA61 (+ VUS_*RNF213*_)	4 IA + 1 aSAH FH neg
		c.287A>G p.Leu96Pro	rs559282550 n.r	VUS_PP3_	1.552% 447/4/28796	32/0.573/n.a./0.183	n.d	IA78 (+ 2VUS_*RNF213*_)	1 giant IA FH pos
								IA84	1 IA FH pos
*PCNT* misZ = -1.33 pLI = 0.0	NM_006031	c.959G > A p.Arg320Lys	rs149844283 194,942	VUS_PM2+BP1+BP4_	0.01048% 26/0/248208	15.60/0.070/0.003/0.097	no impact	IA72	1 IA + 1 aSAH FH neg
		c.2033A>G p.Lys678Arg	rs149623054 265,806	LBV_BP1+BP4_	0.1591% 450/2/282880	8.311/0.026/0.012/0.019	acceptor gain (HSF)	IA85 (+ VUS_*PCNT*_)	4 IA + 1 aSAH FH pos
		c.2839G>C p.Ala947Pro	rs1177229119 n.r	VUS_PM2+PP3+BP1_	0.001595% 4/0/250744	20.5/0.093/0.027/0.669	n.d	IA75	1 IA + 1 aSAH FH neg
		c.4345C>G p.Gln1449Glu	rs139432601 169,656	LBV_BP1+BP4_	0.347% 981/9/282746	14.70/0.134/0.011/0.010	n.d	IA63 (+ ^trans^VUS_*PCNT*_)	2 IA + 1 aSAH FH neg
		c.4354G>A p.Gly1452Arg	rs143796569 169,657	VUS_PP3+BP1+BP4_	0.267% 755/6/282742	22.0/0.227/n.a./0.036	gain of AGGT; donor gain (NG2, BDGP); acceptor gain (HSF)	IA63 (+ ^trans^LBV_*PCNT*_)	2 IA + 1 aSAH FH neg
								IA79 (+ VUS_*RNF213*_)	2 IA FH pos
		c.6404C>T p.Thr2135Met	rs145710874 n.r	VUS_PM2+BP1+BP4_	0.001193% 3/0/2514320	11.77/0.053/0.002/0.078	n.d	IA54 (+ VUS_*THSD1*_)	5 IA FH neg
		c.6739C>T p.His2247Tyr	rs61735812 142,330	LBV_BS4+BP1+BP4_	1.218% 2866/22/235400	1.606/0.038/n.a./0.002	n.d	IA24^d^	1 IA + 1 aSAH FH pos
		c.7652C>T p.Ala2551Val	rs12481791 142,308	VUS_PP3+BP1+BP4_	1.444% 3848/39/266458	22.5/0.109/n.a./0.027	gain of AGGT; donor gain (HSF, NG2, MES)	IA57	4 IA + 2 aSAH FH neg
								IA59	1 IA FH pos
		c.7988G>A p.Arg2663His	rs778334017 430,519	VUS_PM2+BP1+BP4_	0.00115% 2/0/173878	15.38/0.042/0.011/0.467	n.d	IA85 (+ LBV_*PCNT*_)	4 IA + 1 aSAH FH pos
*RNF213* misZ = 2.64 pLI = 0.0	NM_001256071	c.397C>A p.Leu133Met	rs149177904 404,723	VUS_PP2+BP4_	0.1089% 231/1/212142	7.116/0.047/0.006/0.002	n.d	IA78 (+ VUS_*ANGPTL6*_, + VUS_*RNF213*_)	1 giant IA FH pos
		c.626T>A p.Ile209Asn	rs144769597 404,724	VUS_PP2+BP4_	0.1151% 323/1/280624	0.295/0.165/0.017/0.007	n.d	IA78 (+ VUS_*ANGPTL6*_, + VUS_*RNF213*_)	1 giant IA FH pos
		c.8084C>T p.Ala2695Val	rs202096577 n.r	VUS_PP2+BP4_	0.05313% 150/0/282338	24.4/0.325/0.018/0.187	n.d	IA61 (+ VUS_*ANGPTL6*_)	4 IA + 1 aSAH FH neg
		c.14030G>T p.Trp4677Leu	rs61741961 n.r	VUS_PP2+PP3_	1.047% 2962/29/282802	23.4/0.602/n.a./0.088	n.d	IA79 (+ VUS_*PCNT*_)	2 IA FH pos
*THSD1* misZ = 0.49 pLI = 0.0	NM_018676	c.1666G>C p.Gln556Glu	rs148515012 n.r	VUS_BP4_	0.01344% 38/0/282718	25.0/0.228/0.041/0.193	n.d	IA64_S481Nr111_	1 IA FH pos
		c.871C>T p.Glu291Lys	rs41292808 n.r	VUS_BP4_	1.864% 5271/78/282824	12.32/0.090/n.a./0.031	n.d	IA17 (+ VUS_*TMEM132B*_)	1 IA + 1 aSAH FH neg
								IA49	1 IA + 1 AA FH pos
								IA54 (+ VUS_*PCNT*_)	5 IA FH neg
								IA90^e^	3 IA FH pos
		c.592G>C p.Gln198Glu	rs186046951 n.r	VUS_BP4_	0.01097% 31/0/282698	18.67/0.046/0.007/0.071	n.d	IA76	4 IA FH pos
*TMEM132B* misZ = 1.66 pLI = 1.0	NM_052907	c.2737C>A p.Leu913Met	rs61940807 n.r	VUS_PM1+BP4_	0.9920% 2787/52/280952	23.2/0.165/n.a./0.075	n.d	IA17 (+ VUS_*THSD1*_)	1 IA + 1 aSAH FH neg

Variants with MAF ≤ 0.05 (5%) are listed by gene and were detected in 6 out of 8 analyzed genes: *ADAMTS15*, *ANGPTL6*, *PCNT*, *RNF213*, *THSD1* and *TMEM132B;* no variants were identified in *ARHGEF17* and *LOXL2*. For each gene, gnomAD constraint metrics (v2.1) including missense Z score (misZ; intolerance to variation) and pLI score (probability of loss-of-function intolerance) are given. Transcript IDs correspond to the NCBI Reference Sequence (RefSeq) project. Nucleotide and amino acid changes are given according to the indicated transcript ID. Nucleotide numbering uses + 1 as the A of the ATG translation initiation codon in the reference sequence, with the initiation codon as codon 1. Concomitant variants are indicated in the column “Subject” after the patient numbers in parentheses

*aSAH* aneurysmal subarachnoid hemorrhage, *IA* intracranial aneurysm (including unruptured and ruptured IA), *FH pos.* positive family history, *FH neg.* negative family history, *FH n.d.* family history not determined, *n.r.* not reported, *n.a.* not available, *n.d.* not determined

^a^Variant classification: VUS, variant of uncertain significance; LBV, likely benign variant. Classification criteria PM1, PM2, PP3, BS4, BP1 and BP4 are explained in the Supplementary Methods. BP1 (missense variant in a gene for which primarily truncating variants are known to cause disease) was assigned to *PCNT* variants, because truncating variants in *PCNT* are the primary type of pathogenic variants for Microcephalic osteodysplastic primordial dwarfism, type II (MIM #210720), a conditions that is associated with IA. *RNF213* variants were assigned with PP2 (missense variant in a gene that has a low rate of benign missense variation and in which missense variants are a common mechanism of disease), because missense variants in *RNF213* have been associated with susceptibility to Moyamoya disease 2 (*MIM* #607151), a disorder associated with intracranial vascular malformations. References see Supplementary Information

^b^gnomAD, Genome Aggregation Database (v2.1); all MAF, minor allele frequency (total population); het/hom/Σ, number of heterozygous carriers/number of homozygous carriers/total number of analyzed alleles

^c^Details on pathogenicity predictors and respective thresholds [CADD (≥ 20), REVEL (≥ 0.5), M-CAP (≥ 0.025), ClinPred (≥ 0.5)] as well as on splice site predictions with HSF, NetGene2 (NG2), MaxEntScan (MES) and BDGP are outlined in the Supplementary Methods

^d^Variant does not cosegregate with the disease in the family

^e^Variant was identified in further affected family members

In total, 18 unrelated individuals carry variants in reported risk genes (Table 2). Eleven patients (IA24, IA49, IA57, IA59, IA64, IA72, IA75, IA76, IA83, IA84 and IA90) had one variant. Seven patients had concomitant variants: IA63 and IA85 had two variants in *PCNT*; for IA63 these variants were in trans. IA79 showed one variant each in *PCNT* and *RNF213*, IA54 in *PCNT* and *THSD1*, IA61 in *ANGPTL6* and *RNF213*, and IA17 in *THSD1* and *TMEM132B*. Notably, the number of variants had no impact on clinical manifestation. Finally, we detected 3 variants (1 in *ANGPTL6* and 2 in *RNF213*) in patient IA78 (Table 2). Within the analyzed patient cohort, we found 4 recurrent VUS (Table 2): *ANGPTL6* c.287A>G p.(Leu96Pro), *PCNT* c.4354G>A p.(Gly1452Arg) and *PCNT* c.7652C>T p.(Ala2551Val) in 2 patients each; and *THSD1* c.871C>T p.(Glu291Lys) in 4 patients. In case of DNA of further affected and unaffected family members was available, cosegregation with disease was checked. The *PCNT* c.6739C>T p.(His2247Tyr) variant in IA24 did not cosegregate with the disease in the family (Figure S1A), indicating that this variant is not pathogenic. *THSD1* c.871C>T p.(Glu291Lys) in IA90, however, was also identified in 2 affected siblings (Figure S1A), thereby supporting a clinical relevance for this variant.

### Determination of novel putative risk genes

**Fig. 1:**
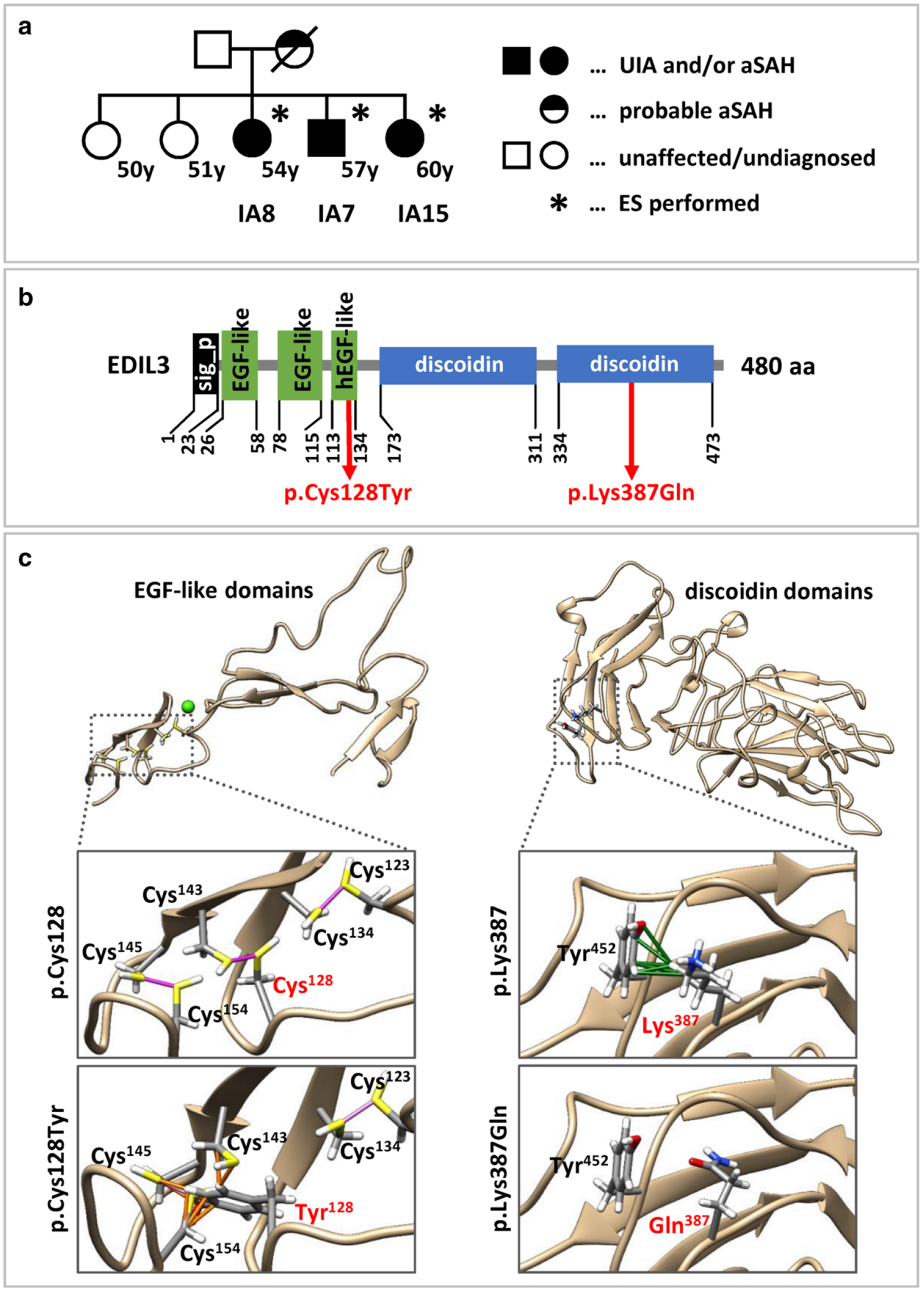
Pedigree for the affected family used for ES, domain structure of EDIL3 and structural impact of EDIL3 amino acid changes. **a** Pedigree. Exome sequenced individuals are marked with an asterisk. Criteria for defining the disease phenotypes (UIA and/or aSAH, probable aSAH) are outlined in the methods section. Age at inclusion is given in years (y). **b** Domain structure of EDIL3 comprising a signal peptide (sig_p, black), three (h)EGF-like domains (green) and 2 discoidin-like domains (blue) is shown relative to the size of the full-length protein. Localization of amino acid changes p.(Cys128Tyr) and p.(Lys387Gln) is indicated. aa, amino acid. **c **Structural impact of EDIL3 p.Cys128Tyr and p.Lys387Gln amino acid changes. Amino acids 128 and 387 and surrounding residues are shown as sticks; side chains are colored by element (hydrogen: white; carbon: grey; oxygen: red; nitrogen: blue; cysteine: yellow). Left column: (h)EGF-like domains of EDIL3 and the structural environment of Cys^128^. Ribbon representations show details of the hEGF-like domain with conserved cysteines that form three and two disulfide bridges (magenta lines) for wild-type (p.Cys128, top) and mutated (p.Cys128Tyr, bottom) EDIL3, respectively. In silico substitution of Cys^128^ for tyrosine resulted in loss of the disulfide bridge with Cys^143^ and overlaps of atomic Van-der Waals (VDW) spheres (orange lines) between Tyr^128^ with cysteines 143, 145 and 154 indicating possible non-covalent clashes. Right column: Discoidin domains of EDIL3 and the structural environment of Lys^387^. Ribbon representations show details of the discoidin domain for wild-type (p.Lys387, top) and mutated (p.Lys387Gln, bottom) EDIL3. For Lys^387^ six VDW contacts with carbons, a nitrogen and a hydrogen of the Tyr^452^ were identified. In silico substitution of Lys^387^ for glutamine resulted in loss of all VDW contacts with Tyr^452^. New VDW contacts or overlaps of Glu^387^ with adjacent residues were not predicted

We performed ES in a family with three affected family members (Fig. 1a). The three siblings share no reportable sequence alteration in known genes associated with vascular/connective tissue disorders (Table S2). From ES data, we extracted potentially pathogenic sequence variants by bioinformatics stratification, biological filter steps and variant prioritization (details are outlined in Supplementary Methods). Briefly, our filtering steps retained homozygous and heterozygous variants if they (i) were present in all affected family members; (ii) were listed neither in the database gnomAD [24], nor in a combined exome dataset of population-based controls from PopGen [25, 26] and of control samples collected in the Institute of Clinical Molecular Biology in Kiel (IKMB-controls); (iii) were predicted to be synonymous variants, missense variants, nonsense variants, frameshift indels or intronic alterations at exon–intron boundaries ranging from − 2 to + 2; and (iv) were predicted to be damaging by CADD, REVEL, M-CAP and/or ClinPred; missense variants with less than two scores above the respective pathogenicity thresholds were excluded; nonsense variants and indels causing premature termination were retained if CADD score was above recommended pathogenicity threshold; synonymous variants were retained if consequences on splicing was suggested by > 1 in silico predictor (Table 3). This stringent filtering pipeline revealed five candidate risk variants for UIA/aSAH in *NEK4, EDIL3, EDNRB, DNAH9* and *GGA3* in this family (Table 3). We classified these variants according to ACMG/AMP guidelines [23], and we defined five VUS (Table 3) including *EDIL3* c.383G>A p.(Cys128Tyr) (Fig. 1b).

**Table 3:** Top ranked putative pathogenic variants shared by three affected siblings

Gene gnomAD constraint metrics	Transcript ID	c. notation p. notation	Classification^a^	Phenotype; MIM number; inheritance	Pathogenicity predictions^b^		Subjects: clinical findings
					CADD/REVEL/M-CAP/ClinPred	Impact on splicing	
*NEK4* misZ = 0.88 pLI = 0.0	NM_003157	c.190A>T p.Asn64Tyr	VUS_PM1+PM2+PP3_	n.r	**23.7**/0.452/**0.113**/**0.984**	n.d	IA7: 2 IA IA8: 1 IA + 1 aSAH IA15: 6 IA
*EDIL3* misZ = 1.16 pLI = 0.0	NM_005711	c.383G>A p.Cys128Tyr	VUS_PM1+PM2+PP3_	n.r	**29.1**/**0.946**/**0.523**/**0.998**	n.d	
*EDNRB* misZ = 1.18 pLI = 0.01	NM_001201397	c.891T>G p.Ser297Arg	VUS_PM2_	Waardenburg syndrome, type 4A; 277580; AD, AR; ABCD syndrome, 600501, AR; Hirschsprung disease, susceptibility to, 2, 600155, AD	**23.7**/0.303/0.020/**0.972**	Gain of ESS site (HSF); no impact (NG2, MES)	
*DNAH9* misZ = − 0.04 pLI = 0.0	NM_001372	c.13304T>C p.Ile4435Thr	VUS_PM2_	Ciliary dyskinesia, primary, 40; 618300; AR	**24.5**/0.203/0.010/**0.983**	n.d	
*GGA3* misZ = − 0.01 pLI = 0.0	NM_138619	c.16G>A p.Gly6Arg	VUS_PM2+PP3_	n.r	**33.0**/0.299/**0.049**/**0.988**	n.d	

Sequence alterations were identified by filtering the exome sequencing data as described in the text. gnomAD constraint metrics (v2.1.1) including missense *Z* score (misZ; intolerance to variation) and pLI score (probability of loss-of-function intolerance) are given. Transcript IDs correspond to the NCBI Reference Sequence (RefSeq) project. Nucleotide and amino acid changes are given according to the indicated transcript ID. Nucleotide numbering uses + 1 as the A of the ATG translation initiation codon in the reference sequence, with the initiation codon as codon 1

*AD* autosomal dominant, *AR* autosomal recessive, *ESS* Exonic Splicing Silencer, *aSAH* aneurysmal subarachnoid hemorrhage, *IA* intracranial aneurysm (including unruptured and ruptured IA), *MIM* Mendelian inheritance in man, *n.d.* not determined, *n.r.* none reported

^a^Variant classification: VUS, variant of uncertain significance; classification criteria PM1, PM2, PP3 and BP4 are explained in the Supplementary Methods. PM1 was assigned to the NEK4 p.Asn64Tyr variant, because of the critical position in the so-called tyrosine-down motif (references see Supplementary Information). PM1 was assigned to the EDIL3 p.Cys128Tyr variant, because of its critical position in an EGF-like domain (see “Discussion”)

^b^Details on pathogenicity predictors and respective thresholds [CADD (≥ 20), REVEL (≥ 0.5), M-CAP (≥ 0.025), ClinPred (≥ 0.5)] as well as on splice site predictions with HSF, NetGene2 (NG2), MaxEntScan (MES) and BDGP are given in the Supplementary Methods. Scores ≥ thresholds are shown in bold numbers

Based on pathogenicity predictions and a strong functional relevance of EDIL3 protein (see “Discussion”, Supplementary Methods and Table S3), we analyzed *EDIL3* coding region in available exome data and by Sanger sequencing in 35 and 37 patients, respectively. We detected a very rare variant classified to be a VUS_PM2+PP3_ in *EDIL3* (c.1159A>C, p.(Lys387Gln)) (Fig. 1b) in two further unrelated patients (IA10 and IA13) both with no reported family history. IA10 showed two intracranial, one carotid and two aortic aneurysms; IA13 had two intracranial aneurysms and aSAH at the age of 35 years. The allele frequency of *EDIL3* c.1159A>C p.(Lys387Gln) is 0.000131 in gnomAD and similarly small with 0.000101 within 5814 healthy control individuals from the German population-based datasets IKMB-controls and PopGen [24–26]. Notably, gnomAD may contain data from cerebrovascular patients and thus the presence of specific variants does not exclude pathogenicity. The variant is predicted to be deleterious by three ensemble pathogenicity classifiers including CADD (score 24.5), REVEL (score 0.723) and M-CAP (score 0.266). We also identified additional variants with MAF ≤ 0.05 in the remaining four candidate risk genes in available exomes of our cohort. However, relatively high MAFs and/or low pathogenicity prediction scores do not support a role for *NEK4*, *EDNRB, DNAH9* and *GGA3* variants in the etiology of UIA/aSAH (Table S4).

To test the association of unknown and very rare *EDIL3* variants (MAF ≤ 0.001) with susceptibility to UIA/aSAH, we compared the numbers of individuals carrying at least one missense variant in this gene among the 73 index case subjects in the present study (3/73 carrier; 4.11%) versus 100 healthy/control individuals with North German ancestry (Hamburg controls: 0/100 carrier; 0.0%) by gene burden and Sequence Kernel Association Tests (SKAT) [27]. We found a significant enrichment in individuals carrying *EDIL3* missense variants with an MAF below 0.001 (0.1%) in our study cohort versus healthy/control individuals (SKAT, *p* = 0.0298; burden test, *p* = 0.0152; Table S5). We obtained even stronger results when comparing allele counts among the 73 index case subjects versus 64,603 non-Finnish European individuals with exome/genome sequences derived from the gnomAD database v2 (326 variants in 64,603 individuals; SKAT, *p* = 2.53e−33; burden test, *p* = 0.0000150; Table S5) [24]. Finally, the cumulated allele frequency of very rare (allele frequencies ≤ 0.001) *EDIL3* missense variants in non-finish Europeans listed in gnomAD (326/64,603) is 0.00505. Compared to this, the cumulative frequency of unknown/very rare variants (i.e. c.383G>A and c.1159A>C) in *EDIL3* in our cohort (3/73; 0.0411) is significantly higher (Fisher’s exact *p* = 0.00624; Table S5).

We explored the structural impacts of the p.(Cys128Tyr) and p.(Lys387Gln) changes using a model for EDIL3 EGF-like domains and discoidin domains, respectively [28, 29]. Molecular replacement of the amino acid Cys^128^ for a tyrosine resulted in (i) loss of the disulfide bridge between amino acid 128 and Cys^143^, and (ii) aberrant intramolecular interactions of Tyr^128^ with cysteines 143, 145 and 154 (Fig. 1c). Replacement of the amino acid Lys^387^ for a glutamine resulted in loss of six intramolecular contacts with Tyr^452^ (Fig. 1c). These data suggest that both p.(Cys128Tyr) and p.(Lys387Gln) induce structural alterations which likely interfere with EDIL3 function.

Taken together, we identified two different *EDIL3* missense variants, c.383G>A p.(Cys128Tyr) and c.1159A>C p.(Lys387Gln), in three affected individuals from one family and in two unrelated patients, respectively. The p.(Cys128Tyr) variant is absent from databases, the p.(Lys387Gln) variant is very rare; this supports the hypothesis that the two variants might be involved in the pathogenesis of the disease. Both alterations are predicted to be damaging by combinational pathogenicity predictors as well as by molecular replacement, and the functional relevance of *EDIL3* for vascular biology is strong (see “Discussion” section). Therefore, *EDIL3* is a valid candidate disease gene for UIA/aSAH.

## Discussion

### Phenotypic aspects

For establishing our study cohort, we focused on patients with a severe clinical phenotype, a family history of UIA and/or SAH and few or no exogenous risk factors [9, 10]. This resulted in a relatively young cohort consisting of patients with proven aSAH or a high number of UIA. As a clinical tool, the PHASES score assists physicians to assess the risk of aneurysm rupture; however, this score does not consider family history and genetic factors. Notably, the scores in our aSAH- and UIA-groups were similar and moderately high. This indicates a moderate sensitivity in a selected cohort of strongly affected patients with a high percentage of positive family history. Thus, incorporating genetic/familial aspects might improve the sensitivity of PHASES scoring system. We found *EDIL3* variants in strongly affected patients with up to six IA. The two sporadic patients had no familial history and few exogenous risk factors for IA formation. One of these patients was diagnosed with three additional extracranial aneurysms and died subsequent to aortic surgery. The co-occurrence of intracranial and extracranial aneurysms in subject IA10 may suggest an overlapping pathogenesis of intracranial and extracranial vascular diseases, as already reported by others [30, 31], and may reflect the general importance of *EDIL3* for vascular integrity.

### Validation of reported risk genes

Continual evaluation of putative risk genes and refinement of reported risk alleles is essential for shaping the genetic etiology of UIA/aSAH [9, 10]. We examined reported risk genes *ADAMTS15*, *ANGPTL6*, *ARHGEF17*, *LOXL2*, *PCNT*, *RNF213*, *THSD1* and *TMEM132B* via exome sequencing in 38 individuals with UIA/aSAH and identified variants with MAF ≤ 0.05 (5%) in 18 of them. Previously, four rare missense variants in *PCNT* exon 38 encoding a coiled coil motif have been identified in affected members of 2 different families and in 3 out of 161 unrelated patients with nontraumatic SAH or UIA (Figure S2A); thus a specific role for this domain in the etiology of IA/SAH has been suggested [8]. We identified *PCNT* variants in 9 patients, which do not cluster in a specific region of the gene (Figure S2A). Two of these variants were predicted to alter splicing, three of these variants are likely benign (Table 2). Neither previously reported *PCNT* missense variants nor those identified in our study localize within a constrained coding region [8, 32]. *PCNT* is a highly mutable gene which is reflected by a very low misZ score (Table 2) [24]. Taken together, *PCNT* remains a good candidate gene for IA/SAH, however, final verification of *PCNT* as a risk gene is still pending. Variants in *RNF213* predispose to Moyamoya disease in diverse ethnicities [33, 34], and rare variants have been recently identified in 25 from 249 patients with IA/SAH in a French Canadian population [4]. Moreover, functional and genetic data strongly support that *RNF* variants are associated with a variety of vascular disorders, as it has been discussed by Zhou et al. (2018) [4]. It was suggested that *RNF213* exon 29 encoding AAA + ATPase domains might be the risk region for IA/SAH (Figure S2B) [4]. We identified four VUS in three patients with IA/SAH; one patient with a giant IA carries two rare *RNF213* variants (Table 2), and the variant c.8084C>T p.(Ala2695Val) in IA61 localizes in the linker region between the two AAA + domains (Figure S3). Moreover, we found a further VUS in *RNF213* (c.10450G>A p.(Gly3484Ser)) in two siblings with SAH but no detectable IAs (Supplementary Results, Table S6 and Figure S1B). Taken together, the plurality of independent evidences and *RNF213* intolerance of variation (misZ = 2.3) indicate that *RNF213* sequence alterations are risk factors for IA/SAH and related disorders. Genetic evidence suggested a role of *THSD1* variants in the pathogenesis of IA/SAH, previously, and missense variants identified in affected individuals clustered in the intracellular protein portion (Figure S2C) [5]. We observed two and one variants in the extracellular and intracellular THSD1 protein portion, respectively (Figure S2C). The *THSD1* c.871C>T p.(Glu291Lys) variant, which has a relatively high MAF (0.01864) was found in four unrelated individuals and it co-segregated with the disease in the family of IA90 (Figure S1A). Taken together, our data do not oppose but rather support a role of *TSHD* variation in the etiology of IA/SAH. For the discussion on variants in *ANGPTL6*, *ADAMTS15*, *TMEM132B*, *ARHGEF17* and *LOXL2* see Supplementary Discussion; overall evidence for an overlap of our data with previous studies on these five genes is very limited [this study, 6, 7, 9–11].

### Genetic and functional aspects on *EDIL3* as an UIA/aSAH risk gene

There is no significant overlap in putative susceptibility genes between various ES-based studies [35], which may reflect a pronounced heterogeneity of IA/SAH pathogenesis. We also aimed to identify genetic risk factors for UIA/aSAH by a family-based ES approach, and we propose *EDIL3* as new risk gene. From the genetic perspective, *EDIL3* is a very good candidate disease gene for UIA and aSAH because we identified likely deleterious variants in affected relatives in a family (Fig. 1a) and, additionally, in two unrelated individuals with UIA/aSAH. Moreover, association tests showed a statistically significant gene burden for *EDIL3*. Functional assessment of EDIL3 protein and the predicted amino acid substitutions (p.(Cys128Tyr), p.(Lys387Gln)) strongly supports this hypothesis (Supplementary Methods, Tables S3 and S4): The mouse homologue Edil3 was identified as a matrix-associated matricellular protein expressed by endothelial cells during embryonic vascular development [36]. Through its interaction with integrin receptors, Edil3 promotes adhesion of endothelial and vascular smooth muscle cells [36, 37]. Both cell types are crucial for wall integrity of blood vessels and angiogenesis [38]. Edil3 is regulated by hypoxia or vascular injury and has been implicated in vascular remodeling during angiogenesis [39–41]. EDIL3 is highly expressed in blood vessels and in brain (Figure S3). Summarized, EDIL3 belongs to a number of epidermal growth factor (EGF) repeat containing genes in the vascular wall, which are essential for wall integrity and remodeling [37]. Amino acid changes p.(Cys128Tyr) and p.(Lys387Gln) affect highly conserved domains. EDIL3 has 3 N-terminal EGF-like domains, followed by two discoidin domains (Fig. 1b) [36]. The EGF modules are stabilized by six conserved cysteines forming 3 disulfide bonds in the pattern of 1–3, 2–4, and 5–6 (Fig. 1c) [28]. The p.(Cys128Tyr) change affects the 2nd Cysteine in the 3rd calcium-binding EGF-like domain, which likely compromises the shape and function of the small domain (Fig. 1c). Accordingly, mutations of highly conserved cysteines in calcium-binding EGF-like domains play a seminal role in several diseases such as the Marfan syndrome [42]. The p.(Lys387Gln) change alters the 2nd discoidin domain (Fig. 1c), a structural entity that is found in many extracellular and membrane proteins involved in cellular adhesion, migration, or aggregation events, and associated with organogenesis (vasculogenesis and angiogenesis) [43]. The exact role of Lysine 387 that lies within a β-sheet of the discoidin domain has not yet been documented, however modelling of p.Lys387Gln suggested strong effects on the domain structure (Fig. 1c). Notably, it has been shown that depletion of EDIL3 disrupts TGFβ signaling pathways [44]. Dysregulation of this signaling pathway is widely accepted to be central in the pathogenesis of hereditary aortopathies [45]. Numerous familial linkage and GWAS reporting on potential gene loci for IAs have been summarized elsewhere [18, 21]. A genome-wide-linkage/haplotype-association analysis revealed a strong evidence for a linkage of IAs to the chromosomal region 5q14.3 with a p value of 0.001 for marker D5S428; this DNA marker localizes ca. 1 Mb distal from *EDIL3* [46].

## Conclusions

ES studies in multiplex families produce reasonable candidate disease genes for UIA/aSAH. To validate or discard putative disease genes, large cohorts of UIA/aSAH families and sporadic patients are needed for future next-generation sequencing studies; thus, collaboration between research groups with substantial cohorts of patients will be essential. Based on the premise that there is pathophysiological overlap, ES data of individuals with other vascular diseases such as thoracic/abdominal aortic aneurysm could also be included. Definitive identification of genetic risk factors is highly desirable, as it will contribute to develop future tests to assess the risk of SAH in a routine clinical setting.

## Electronic supplementary material

Below is the link to the electronic supplementary material.

Supplementary file1 (DOCX 57 kb)

** Figure S1. **Pedigrees for families of subjects IA24 and IA90 as well as SAH45 and SAH39. Exome sequenced individuals are marked with an asterisk. Criteria for defining the disease phenotypes (UIA and/or aSAH, probable aSAH) are outlined in the methods section. Age at inclusion is given in years (y). Individuals positive (red label) and negative (green label) for the respective sequence variant are indicated. (TIF 195 kb)

** Figure S2. **Schematic representation of proteins previously associated with IA/SAH. Distribution of putative disease-associated amino acid changes and protein domains/motifs with position specifications are shown. Figure were designed with the Pfam database. aa, amino acids.** A**. PCNT variants and protein domains and motifs. Variants identified in this study (red letters) and variants reported in Lorenzo-Betancor *et al*. (2018) (black letters) are given. **B**. RNF213 functional variants found in individuals with IA and Moyamoya disease (black and grey letters, respectively); figure was adapted from Zhou et al. (2016). Five variants found in the current study are marked in red. **C**. THSD1 variants and protein domains and motifs. Variants identified in this study (red letters) and variants reported in Santiago-Sim *et al*. (2016) (black letters) are given. **D**. ANGPTL6 variants and protein domains and motifs. Variants identified in this study (red letters) and variants reported in Bourcier *et al*. (2018) (black letters) are given. **E**. ADAMTS15 variants and protein domains and motifs. Variants identified in this study (red letters) and variants reported in Yan *et al*. (2015) (black letters) are given. **F**. TMEM132B variants and protein domains and motifs. Variants identified in this study (red letters) and variants reported in Farlow *et al*. (2015) (black letters) are given. (TIF 864 kb)

** Figure S3. **Tissue-specific gene expression of *EDIL3*. This figure was obtained from the Genotype-Tissue Expression (GTEx) Portal on 02/19/2019 and dbGaP accession number phs000424.v7.p2. The GTEx Project was supported by the Common Fund of the Office of the Director of the National Institutes of Health, and by NCI, NHGRI, NHLBI, NIDA, NIMH, and NINDS. (TIF 1536 kb)
