# Supplementary material for: Exome sequencing in 38 patients with intracranial aneurysms and subarachnoid hemorrhage
Source: J Neurol. 2020 May 4;267(9):2533–45. doi: 10.1007/s00415-020-09865-6 (PMC7419486; doi:10.1007/s00415-020-09865-6)
Supplement: Supplementary file 1 — Supplementary file1 (DOCX 57 kb) [file 415_2020_9865_MOESM1_ESM.docx]

**Supplementary Material**

**Sauvigny et al., (2019) Exome sequencing in 38 patients with intracranial aneurysms and subarachnoid hemorrhage**

**SuppLementaRY Introduction**

In the **Introduction** we refer on eight different papers reporting on alterations in *ANGPTL6*, *RNF213*, *THSD1*, *ARHGEF17*, *PCNT* *ADAMTS15*, *TMEM132B* and *LOXL2* as (putative) genetic risk factors for this disease [1-8]. However, there is a variation in the study design, academic depth and, hence, scientific validity among these papers: The *ANGPTL6* study is based on exome sequencing, familial segregation analysis, gene resequencing, identity-by-descent analysis, gene burden testing and functional analysis [3]. Risk alleles in *RNF13* were identified by exome sequencing, SNP genotyping, statistical and population analysis as well as functional analysis [1]. The identification of *THSD1* as risk gene based on linkage analysis, exome sequencing, gene resequencing, statistical calculation and functional analysis [2,9]. *ARHGEF17* variants were identified and verified by genome sequencing, exome sequencing, identity-by-descent analysis, statistical and population analysis and functional studies [4]. The description of *PCNT* as susceptibility gene for IA/SAH based on exome sequencing, gene sequencing, haplotype analysis [5]. In the study resulting in *ADAMTS15* as a risk gene, exome sequencing, gene resequencing, statistical calculations and functional analysis were performed [7]. IA/SAH-associated *TMEM132B* variants were found by exome sequencing, linkage analysis and gene expression analysis [10,6,11]. Finally, for the description of *LOXL2* as putative risk factor exome sequencing and gene resequencing was done [8]. Taken together, all these studies include exome sequencing as a key technology and common denominator for the identification of novel susceptibility genes for IA/SAH.

**SuppLementaRY Methods**

**Selection criteria and constraints of individuals for exome sequencing**

38 patients including three family members were exome sequenced. Selection was based on: (**i**) Written informed consent of probands for exome sequencing; if there was no such consent, we obtained consent for Sanger sequencing of candidate genes according to the national legal regulations (i.e. the German Genetic Diagnosis Act [GenDG]). For several patients (e.g. IA3, IA9, IA11 etc.; **Table S1**) we were not allowed to perform exome sequencing. (**ii**) We gave priority to affected individuals with a positive family history (e.g. IA7, IA59, IA64 etc.; **Table S1**). (**iii**) If family history was negative, only substantially affected patients with ≥2 UIA or ≥1 UIA and aSAH were included (e.g. IA6, IA13, IA58 etc.; **Table S1**). (**iv**) Finally, we had financial restrictions that kept us from exome sequencing of some clinically highly relevant patients (e.g. IA11, IA34, IA106 etc. **Table S1**). We exome sequenced five patients (IA49, IA59, IA64, IA78 and IA84; **Table S1**) with 1 UIA but without aSAH; the reasons for inclusion of these five patients are given in the footnotes of **Table S1**.

**Exome sequencing, variant calling, filtering strategies and variant prioritization**

***Exome sequencing***

Genomic DNA was extracted from peripheral blood samples using standard procedures. Exome sequencing (ES) of patients IA6, IA13, IA18, IA40, IA47, IA49-50, IA53-54, IA57-58, IA60-61, IA63, IA70-73 as well as SAH39, SAH41 and SAH45 was performed by the Cologne Center for Genomics (CCG, Cologne, Germany): DNAs were subjected to the standard protocol SureSelectXT Automated Target Enrichment for Illumina paired-end multiplexed sequencing using the Bravo automated liquid handling platform (Agilent, Santa Clara, CA). DNAs were sheered by ultrasonic waves and nearly all of the coding regions including surrounding intronic sequences were isolated and enriched from DNA samples (SureSelect Human All Exon V6). Captured libraries were sequenced on a HiSeq platform (Illumina) in 2x75 bp paired-end mode. ES of patients IA7, IA8, IA15, IA17, IA24, IA59, IA64, IA69, IA74-79, IA83-85, IA90, IA92 and IA95 as well as of 100 healthy control individuals (Hamburg controls) was performed at the Center for Genomics and Transcriptomics (CeGaT, Tübingen, Germany): coding regions including surrounding intronic sequences were isolated and enriched from DNA samples using the in-solution technology SureSelectXT Human All Exon V6 (Agilent, Santa Clara, CA). Captured libraries were sequenced on a HiSeq platform (Illumina) in 2x100 bp paired-end mode. On average, 89.28% and 94.04% of targeted regions were covered 30x and 20x, respectively. Trimmomatic v.0.36 [12] was used to trim sequences of sequencing adapters and suffixes of low quality (Phred quality score below 10).

***Variant calling***

Variant calling was performed following the Genome Analysis Toolkit's (GATK v.3.8.0) best practice recommendations [13]. Briefly, the trimmed reads were first aligned to the human reference genome (UCSC GRCh37/hg19) using the Burrows-Wheeler Aligner BWA-MEM v.0.7.15 [14] and duplicate reads were removed with Picard tools v.2.8.3 (http://broadinstitute.github.io/picard). GATK was used for base quality score recalibration, calling variants using the HaplotypeCaller, joint genotyping, and variant quality score recalibration. AnnoVar v.2016-02-01 [15] was employed to annotate alterations using information from public databases (dbSNP147, Exome Variant Server, Genome Aggregation Database (gnomAD) and 1000 Genomes Project).

***Filtering strategy (for family-based disease gene identification)***

Pedigrees of UIA/aSAH families suggested that UIA/aSAH is either a Mendelian disorder (a variant in a gene explains a significant part of the phenotype) or a monogenic disorder (a variant explains the complete phenotype) [6,7,2,1]. Therefore, we searched for unknown variants with anticipated significant effect size shared by affected family members (**Figure 1A**). Because of reduced penetrance and late onset of UIA/aSAH, non-affected family members were not included as mutation-negative controls.

***Variant prioritization***

ES data were screened for sequence variants in known disease genes for vascular/connective tissue disorders (**Table S2**). The presence of possibly pathogenic variants in these genes was assessed and excluded on the basis of allele frequencies (≤ 0.05 according to gnomAD [16] and popfreq_max databases [17,18]), genetic impact (missense, nonsense, coding indels and intronic alterations at exon-intron boundaries ranging from -2 to +2) and predicted pathogenicity (using CADD, REVEL, M-CAP and ClinPred scoring systems; pathogenicity thresholds are given below).

For the validation of previously reported putative IA/SAH risk genes, ES data were filtered for homozygous and heterozygous variants with minor allele frequency (MAF) ≤ 0.05 (5%) in the databases gnomAD [16] and popfreq_max [17,18] and the 8 genes *ADAMTS15*, *ANGPTL6*, *ARHGEF17*, *LOXL2*, *PCNT*, *RNF213*, *THSD1* and *TMEM132B* were further evaluated. The minor allele frequency (MAF) refers to the frequency at which the second most common allele occurs in a given population. For the identification of novel disease candidate genes (family-based approach), ES data were filtered for variants not listed in the a.m. databases and all genes were included in further analysis.

Only variants with high genetic impact (missense, nonsense, frameshift indels and intronic alterations at exon-intron boundaries ranging from -2 to +2) were retained. For confirmation and interpretation of variant calls, genomic regions of interest were visualized with the Integrative Genomics Viewer IGV v.2.3.25 [25,26]. Mutational effects were predicted with CADD (Combined Annotation Dependent Depletion), REVEL (Rare exome variant ensemble learner), M-CAP (Mendelian Clinically Applicable Pathogenicity) and ClinPred (Prediction Tool to Identify Disease-Relevant Nonsynonymous Single-Nucleotide Variants) scoring systems; pathogenicity thresholds were selected according the respective authors’ recommendations: CADD (≥20), REVEL (≥0.5), M-CAP (≥0.025), ClinPred (≥0.5) [27-30]. For some alleles M-CAP scores are not available because M-CAP scores only rare (≤ 1% allele frequency) missense variants based on the Ensembl build 75 gene set. Splice site predictors were applied if a sequence alteration (**i**) localized near exon intron borders, or (**ii**) might result in the formation of a novel consensus splicesite: HSF, Human Splicing Finder [31]; NetGene2 (NG2), neural network predictions of splice sites [32]; MaxEntScan (MES), Maximum Entropy Modeling of Short Sequence Motifs [33]; BDGP, Berkeley Drosophila Genome Project [34].

For family-based gene identification approach, variants were ranked according to their individual scores; the respective genes and their encoded proteins were reviewed regarding their functional relevance in vascular biology by using various databases and repositories. In detail, genes with GO (Gene Ontology) terms [35], that were hypothesized to be involved in UIA/aSAH pathophysiology (i.e. vascular biology (e.g. GO:0001944, GO:0001570, GO:0003018, GO:0101023, GO:0001525), extracellular matrix components and homeostasis (e.g. GO:0031012, GO:0050840, GO:0005581, GO:0005604, GO:0051541), cell adhesion or cytoskeleton (e.g. GO:0031589, GO:0098609, GO:0090675, GO:0005925, GO:0022610, GO:0005856), TGFβ or SMAD signal transduction (e.g. GO:0035583, GO:0038105, GO:0038044, GO:2001203, GO:2001201, GO:0060395), smooth muscle or endothelial cell biology (e.g. GO:0051145, GO:0048659, GO:0071670, GO:0061302, GO:0014909; GO:0043542)) were prioritized and attributed with “Gene ontology positive” (“O+”; **Table S3**). We selected these GO terms resting on the given histology of blood vessels and knowledge about the pathophysiology of hereditary aortopathy [36]. Furthermore, we checked, if encoded proteins physically interact with extracellular matrix components, TGFβ or SMAD signaling molecules, cytoskeletal regulators/components or proteins known to be strongly involved in vascular biology or encoded by known vascular disease genes; for this we used the BioGRID [37,38] and IntAct [39] interaction databases; the respective genes were attributed with “interaction positive” (“I+”; **Table S3**). UniProt [40] and Pfam databases [41] were used to check if the detected alterations affect annotated protein domains or motives or posttranslationally modified amino acids; if this was the case, genes attributed with “amino acid positive” (“A+”; **Table S3**). The Human Protein Atlas [42], the Genevisible database [43] and/or the Genotype-Tissue Expression database (GTEx; https://gtexportal.org/home/) were used to review if the expression of the gene of interest is enhanced in smooth muscle or endothelial cells; such genes were attributed with “expression positive” (“E+”; **Table S3**).

Minor allele frequencies (MAFs) of top ranked putative pathogenic variants in population-specific controls were determined by accessing exome data from the database PopGen and the in-house exome dataset from the Institute of Clinical Molecular Biology in Kiel, Germany (IKMB-controls). PopGen includes 1,198 healthy control individuals of Northwestern German descent [44,45], the IKMB-controls dataset includes exomes from 4,616 healthy control individuals of German descent.

Known disease-associated genes were assessed by using OMIM online catalog and the PubMed search engine [46,47].

**Variant classification**

Variants passing these filters and scoring systems were assigned to pathogenicity classes according the American College of Medical Genetics and Genomics and the Association for Molecular Pathology (ACMG/AMP) standards and guidelines: PV, pathogenic variant; LPV, likely pathogenic variant; VUS, variant of uncertain significance; LBV, likely benign variant; pathogenic criteria were weighted as very strong (PVS1), strong (PS1–4), moderate (PM1–6), or supporting (PP1–4); benign criteria were weighted as stand-alone (BA1), strong (BS1–4), or supporting (BP1–5) [48,49]. We assigned PP3 supporting weight to a variant only if at least three out of four metapredictors calculated a pathogenicity score above the respective thresholds or if at least two out of the three splice site predictors suggested consequences on splicing (**Tables 2** and **3**). If M-CAP scores were not available, we assigned PP3 supporting weight to a variant only if at least two out of three metapredictors calculated a pathogenicity score above the respective thresholds. BP4 was assigned if fewer than 2 metapredictors calculated a pathogenicity score above the respective thresholds or if fewer than 2 splice site predictors suggested consequences on splicing (**Tables 2** and **3**).

BP1 (missense variant in a gene for which primarily truncating variants are known to cause disease) was assigned to *PCNT* variants (**Table 2**), because truncating variants in *PCNT* are the primary type of pathogenic variants for Microcephalic osteodysplastic primordial dwarfism, type II (MIM #210720), a conditions that is associated with IA [50,5]. *RNF213* variants were assigned with PP2 (missense variant in a gene that has a low rate of benign missense variation and in which missense variants are a common mechanism of disease) (**Table 2**), because missense variants in this gene have been associated with susceptibility to Moyamoya disease 2 (MIM #607151), a disorder associated with intracranial vascular malformations [51,1]. PM1 was assigned to the *NEK4* p.Asn64Tyr variant (**Table 3**), because of the critical position in the so-called tyrosine-down motif [52,53]. PM1 was assigned to the *EDIL3* p.Cys128Tyr variant (**Table 3**), because of its critical position in an EGF-like domain (see discussion) [54].

**Variant validation and mutation analysis**

Available exomes of 35 patients with UIA/aSAH were screened for variants with MAF ≤ 0.05 (5%) in the five top ranked disease candidate genes (**Table S3**). Genetic impact, allele frequency and predicted pathogenicity of detected sequence variants were assessed as described in *variant prioritization* (see above). Sanger sequencing was applied for mutation screening of the candidate disease gene *EDIL3* (NM_005711.4) in our rest cohort consisting of 38 patients with UIA/aSAH. Primer pairs designed to amplify the coding exons and intron boundaries as well as PCR conditions are available upon request. Amplicons were directly sequenced using ABI BigDye Terminator Sequencing Kit (Applied Biosystems, Darmstadt, Germany) and an automated capillary sequencer (ABI 3500; Applied Biosystems). Sequence electropherograms were analyzed using the Sequence Pilot software (JSI Medical Systems, Kippenheim, Germany). Genetic impact, allele frequency and predicted pathogenicity of detected sequence variants were assessed as described in *variant prioritization* (see above).

**Presentation of clinical data**

Standard definitions were used for patient variables and outcomes. Categorical variables are presented as percentages, and continuous variables are expressed as mean ± SD with range throughout the manuscript.

**Statistical analysis**

Gene association was calculated by using original weighted linear kernel SNP-set (Sequence) Kernel Association Test (SKAT) with the application of the exact method to compute the p-value (SKAT R package version 1.3.2.1 and R version 3.5.3) [55-58]; gene burden testing is also covered by this software package. The Fisher’s exact test was used to test whether there is an association between very rare (allele frequencies ≤ 0.001 (0.1%) [59]) missense variants in *EDIL3* and the disease status (control vs. study cohort with UIA/aSAH). Control population data was provided by gnomAD [16]. Notably, gnomAD may contain data from cerebrovascular patients and thus the presence of specific variants does not exclude pathogenicity. Fisher’s exact test was performed with R version 3.5.2 [58].

**Molecular Modelling**

Molecular graphics were developed with UCSF Chimera, and for molecular replacement and analysis, UCSF Chimera build-in tools were used [60]. The most favorable torsion of the side chains predicted by UCSF Chimera is shown (**Figure 1**). For modelling the structural environment of Cys^128^, the crystallographic structure of the EDIL3 EGF domains in complex with a calcium ion (light green) was used as a template (PDB ID 4D90; MMDB ID: 100114) [54]. For modelling the structural environment of Lys^387^, the crystallographic structure of the epithelial discoidin domain-containing receptor 1 (DDR1) was used as a template (PDB ID 4AG4; MMDB ID 98867) [61]. Covalent disulfide bridges as well as contacts between or overlaps of atomic Van-der Waals (VDW) spheres within a 5.0-Å range were identified by using UCSF Chimera contacts finding tool [60]. Contacts between atoms separated by 2 bonds or less as well as intra-residue contacts were ignored.

**SUPPLEMENTARY Results**

**Genetic analysis of top ranked disease candidate genes**

We explored the available exomes of 35 patients with UIA and/or aSAH as well as of 3 patients with SAH but no detectable IAs for variants with MAF ≤ 0.05 (5%) in the five top ranked disease candidate genes (**Table S3**). We identified one additional variant in *NEK4*, one additional variant in *EDIL3*, two additional variants in *EDNRB*, three additional variants in *DNAH9* and two additional variants in *GGA3*. **Table S4** shows both the familial variants in five top ranked candidate disease genes and all additional variants in these genes. Relatively high MAFs and/or low pathogenicity prediction scores do not support a role for *NEK4*, *EDNRB,* *DNAH9* and GGA3 variants in the etiology of UIA/aSAH (**Table S4**). In contrast, the minor allele frequency of *EDIL3* c.1159A>C is very low (0.000131 in gnomAD [16], 0.000101 in PopGen/IKMB-controls [44,45]) and EDIL3 p.(K387Q) is predicted to be deleterious by three out of four ensemble pathogenicity classifiers (**Table S4**). These data and the strong functional relevance of EDIL3 protein in vascular biology prompted us to pursue *EDIL3* as top candidate disease gene.

**Association tests**

We tested the association of unknown and very rare *EDIL3* variants with susceptibility to UIA/aSAH by performing SNP-set (Sequence) Kernel Association Test (SKAT), gene burden test and Fisher’s Exact test (**Table S5**).

**Genetic analysis of reported risk genes in patients with SAH**

In addition to our cohort of patients exclusively with UIA and/or aneurysmal SAH, we also performed ES in two siblings and one sporadic patient with SAH but without angiographic aneurysm evidence. Exome data were analyzed for variants with MAF ≤ 0.05 in the reported risk genes *ADAMTS15*, *ANGPTL6*, *ARHGEF17*, *LOXL2*, *PCNT*, *RNF213*, *THSD1* and *TMEM132B*. Whereas we did not detect a reportable sequence variant in the sporadic patient SAH41, we identified a VUS, *RNF213* c.10450G>A p.(Gly3484Ser), in two siblings (SAH45 and SAH39) with angiogram-negative SAH (no IA detected) (**Table S6**, **Figure S1B**). This data are consistent with the hypothesis that specific *RNF13* variants are risk factors for cerebrovascular disorders.

**SUPPLEMENTARY Discussion**

**Validation of reported risk genes**

We examined reported risk genes *ADAMTS15*, *ANGPTL6*, *ARHGEF17*, *LOXL2*, *PCNT*, *RNF213*, *THSD1* and *TMEM132B,* and identified variants with MAF ≤ 0.05 (5%) in 18 unrelated individuals with UIA/aSAH. Previously, four rare/unknown coding variants including a nonsense, 2 missense and a frameshift indel in *ANGPTL6* have been described in 6 families with IA [3]; here we identified 2 further missense variants in 3 individuals with UIA/aSAH. *ANGPTL6* variants do not cluster in a specific protein region nor constitute a specific mutation type, such as exclusively truncating or missense variants (**Figure S2D**). Thus, the association of rare *ANGPTL6* variants with IA needs further verification. ADAMTS19 p.E133Q was aggregated significantly in familial IA cases in the Japanese population [7]. We identified only one VUS in *ADAMTS15* in an affected individual within our cohort (**Figure S2E**); this variant alters the last nucleotide in exon 3 and was predicted to affect splicing suggesting LOF (loss-of-function) consequences (**Table 2**). However, *ADAMTS15* is tolerant of LOF variation (pLI score=0, **Table 2**). Taken together, to substantiate a role in the pathogenesis of IA/SAH, *ADAMTS15* needs further replication studies. Based on exome sequencing and expression studies *TMEM132B* has been suggested as a risk gene for IA/SAH [6]. We found only one VUS with a relatively high MAF (0.0099) that localizes in an uncharacterized C-terminal domain (**Figure S2F**). Thus, our data do not indicate that *TMEM132B* variants are risk factors for IA/SAH. Finally, our data do not provide further evidence for *ARHGEF17* and *LOXL2* to be susceptibility genes for IA/SAH. For the discussion on variants in *RNF213*, *PCNT and* *THSD1* see the **Discussion** section in the main manuscript.

**SuppLementARY REFERENCES**

1. Zhou S, Ambalavanan A, Rochefort D, Xie P, Bourassa CV, Hince P, Dionne-Laporte A, Spiegelman D, Gan-Or Z, Mirarchi C, Zaharieva V, Dupre N, Kobayashi H, Hitomi T, Harada K, Koizumi A, Xiong L, Dion PA, Rouleau GA (2016) RNF213 Is Associated with Intracranial Aneurysms in the French-Canadian Population. Am J Hum Genet 99 (5):1072-1085. doi:10.1016/j.ajhg.2016.09.001

2. Santiago-Sim T, Fang X, Hennessy ML, Nalbach SV, DePalma SR, Lee MS, Greenway SC, McDonough B, Hergenroeder GW, Patek KJ, Colosimo SM, Qualmann KJ, Hagan JP, Milewicz DM, MacRae CA, Dymecki SM, Seidman CE, Seidman JG, Kim DH (2016) THSD1 (Thrombospondin Type 1 Domain Containing Protein 1) Mutation in the Pathogenesis of Intracranial Aneurysm and Subarachnoid Hemorrhage. Stroke 47 (12):3005-3013. doi:10.1161/STROKEAHA.116.014161

3. Bourcier R, Le Scouarnec S, Bonnaud S, Karakachoff M, Bourcereau E, Heurtebise-Chretien S, Menguy C, Dina C, Simonet F, Moles A, Lenoble C, Lindenbaum P, Chatel S, Isidor B, Genin E, Deleuze JF, Schott JJ, Le Marec H, Group IS, Loirand G, Desal H, Redon R (2018) Rare Coding Variants in ANGPTL6 Are Associated with Familial Forms of Intracranial Aneurysm. Am J Hum Genet 102 (1):133-141. doi:10.1016/j.ajhg.2017.12.006

4. Yang X, Li J, Fang Y, Zhang Z, Jin D, Chen X, Zhao Y, Li M, Huan L, Kent TA, Dong JF, Jiang R, Yang S, Jin L, Zhang J, Zhong TP, Yu F (2018) Rho Guanine Nucleotide Exchange Factor ARHGEF17 Is a Risk Gene for Intracranial Aneurysms. Circ Genom Precis Med 11 (7):e002099. doi:10.1161/CIRCGEN.117.002099

5. Lorenzo-Betancor O, Blackburn PR, Edwards E, Vazquez-do-Campo R, Klee EW, Labbe C, Hodges K, Glover P, Sigafoos AN, Soto AI, Walton RL, Doxsey S, Bober MB, Jennings S, Clark KJ, Asmann Y, Miller D, Freeman WD, Meschia J, Ross OA (2018) PCNT point mutations and familial intracranial aneurysms. Neurology 91 (23):e2170-e2181. doi:10.1212/WNL.0000000000006614

6. Farlow JL, Lin H, Sauerbeck L, Lai D, Koller DL, Pugh E, Hetrick K, Ling H, Kleinloog R, van der Vlies P, Deelen P, Swertz MA, Verweij BH, Regli L, Rinkel GJ, Ruigrok YM, Doheny K, Liu Y, Broderick J, Foroud T, Investigators FIAS (2015) Lessons learned from whole exome sequencing in multiplex families affected by a complex genetic disorder, intracranial aneurysm. PLoS One 10 (3):e0121104. doi:10.1371/journal.pone.0121104

7. Yan J, Hitomi T, Takenaka K, Kato M, Kobayashi H, Okuda H, Harada KH, Koizumi A (2015) Genetic study of intracranial aneurysms. Stroke 46 (3):620-626. doi:10.1161/STROKEAHA.114.007286

8. Wu Y, Li Z, Shi Y, Chen L, Tan H, Wang Z, Yin C, Liu L, Hu J (2018) Exome Sequencing Identifies LOXL2 Mutation as a Cause of Familial Intracranial Aneurysm. World Neurosurg 109:e812-e818. doi:10.1016/j.wneu.2017.10.094

9. Santiago-Sim T, Depalma SR, Ju KL, McDonough B, Seidman CE, Seidman JG, Kim DH (2009) Genomewide linkage in a large Caucasian family maps a new locus for intracranial aneurysms to chromosome 13q. Stroke 40 (3 Suppl):S57-60. doi:10.1161/STROKEAHA.108.534396

10. Foroud T, Investigators FIAS (2013) Whole exome sequencing of intracranial aneurysm. Stroke 44 (6 Suppl 1):S26-28. doi:10.1161/STROKEAHA.113.001174

11. Foroud T, Sauerbeck L, Brown R, Anderson C, Woo D, Kleindorfer D, Flaherty ML, Deka R, Hornung R, Meissner I, Bailey-Wilson JE, Langefeld C, Rouleau G, Connolly ES, Lai D, Koller DL, Huston J, 3rd, Broderick JP, Familial Intracranial Aneurysm Study I (2009) Genome screen in familial intracranial aneurysm. BMC Med Genet 10:3. doi:10.1186/1471-2350-10-3

12. Bolger AM, Lohse M, Usadel B (2014) Trimmomatic: a flexible trimmer for Illumina sequence data. Bioinformatics 30 (15):2114-2120. doi:10.1093/bioinformatics/btu170

13. McKenna A, Hanna M, Banks E, Sivachenko A, Cibulskis K, Kernytsky A, Garimella K, Altshuler D, Gabriel S, Daly M, DePristo MA (2010) The Genome Analysis Toolkit: a MapReduce framework for analyzing next-generation DNA sequencing data. Genome research 20 (9):1297-1303. doi:10.1101/gr.107524.110

14. Li H, Durbin R (2009) Fast and accurate short read alignment with Burrows-Wheeler transform. Bioinformatics 25 (14):1754-1760. doi:10.1093/bioinformatics/btp324

15. Yang H, Wang K (2015) Genomic variant annotation and prioritization with ANNOVAR and wANNOVAR. Nat Protoc 10 (10):1556-1566. doi:10.1038/nprot.2015.105

16. Lek M, Karczewski KJ, Minikel EV, Samocha KE, Banks E, Fennell T, O'Donnell-Luria AH, Ware JS, Hill AJ, Cummings BB, Tukiainen T, Birnbaum DP, Kosmicki JA, Duncan LE, Estrada K, Zhao F, Zou J, Pierce-Hoffman E, Berghout J, Cooper DN, Deflaux N, DePristo M, Do R, Flannick J, Fromer M, Gauthier L, Goldstein J, Gupta N, Howrigan D, Kiezun A, Kurki MI, Moonshine AL, Natarajan P, Orozco L, Peloso GM, Poplin R, Rivas MA, Ruano-Rubio V, Rose SA, Ruderfer DM, Shakir K, Stenson PD, Stevens C, Thomas BP, Tiao G, Tusie-Luna MT, Weisburd B, Won HH, Yu D, Altshuler DM, Ardissino D, Boehnke M, Danesh J, Donnelly S, Elosua R, Florez JC, Gabriel SB, Getz G, Glatt SJ, Hultman CM, Kathiresan S, Laakso M, McCarroll S, McCarthy MI, McGovern D, McPherson R, Neale BM, Palotie A, Purcell SM, Saleheen D, Scharf JM, Sklar P, Sullivan PF, Tuomilehto J, Tsuang MT, Watkins HC, Wilson JG, Daly MJ, MacArthur DG, Exome Aggregation C (2016) Analysis of protein-coding genetic variation in 60,706 humans. Nature 536 (7616):285-291. doi:10.1038/nature19057

17. Genomes Project C, Auton A, Brooks LD, Durbin RM, Garrison EP, Kang HM, Korbel JO, Marchini JL, McCarthy S, McVean GA, Abecasis GR (2015) A global reference for human genetic variation. Nature 526 (7571):68-74. doi:10.1038/nature15393

18. Tennessen JA, Bigham AW, O'Connor TD, Fu W, Kenny EE, Gravel S, McGee S, Do R, Liu X, Jun G, Kang HM, Jordan D, Leal SM, Gabriel S, Rieder MJ, Abecasis G, Altshuler D, Nickerson DA, Boerwinkle E, Sunyaev S, Bustamante CD, Bamshad MJ, Akey JM, Broad GO, Seattle GO, Project NES (2012) Evolution and functional impact of rare coding variation from deep sequencing of human exomes. Science 337 (6090):64-69. doi:10.1126/science.1219240

19. Capuano A, Bucciotti F, Farwell KD, Tippin Davis B, Mroske C, Hulick PJ, Weissman SM, Gao Q, Spessotto P, Colombatti A, Doliana R (2016) Diagnostic Exome Sequencing Identifies a Novel Gene, EMILIN1, Associated with Autosomal-Dominant Hereditary Connective Tissue Disease. Hum Mutat 37 (1):84-97. doi:10.1002/humu.22920

20. Kuang SQ, Medina-Martinez O, Guo DC, Gong L, Regalado ES, Reynolds CL, Boileau C, Jondeau G, Prakash SK, Kwartler CS, Zhu LY, Peters AM, Duan XY, Bamshad MJ, Shendure J, Nickerson DA, Santos-Cortez RL, Dong X, Leal SM, Majesky MW, Swindell EC, Jamrich M, Milewicz DM (2016) FOXE3 mutations predispose to thoracic aortic aneurysms and dissections. The Journal of clinical investigation 126 (3):948-961. doi:10.1172/JCI83778

21. Lee VS, Halabi CM, Hoffman EP, Carmichael N, Leshchiner I, Lian CG, Bierhals AJ, Vuzman D, Brigham Genomic M, Mecham RP, Frank NY, Stitziel NO (2016) Loss of function mutation in LOX causes thoracic aortic aneurysm and dissection in humans. Proc Natl Acad Sci U S A 113 (31):8759-8764. doi:10.1073/pnas.1601442113

22. Guo DC, Gong L, Regalado ES, Santos-Cortez RL, Zhao R, Cai B, Veeraraghavan S, Prakash SK, Johnson RJ, Muilenburg A, Willing M, Jondeau G, Boileau C, Pannu H, Moran R, Debacker J, GenTac Investigators NHL, Blood Institute Go Exome Sequencing P, Montalcino Aortic C, Bamshad MJ, Shendure J, Nickerson DA, Leal SM, Raman CS, Swindell EC, Milewicz DM (2015) MAT2A mutations predispose individuals to thoracic aortic aneurysms. Am J Hum Genet 96 (1):170-177. doi:10.1016/j.ajhg.2014.11.015

23. Salo AM, Cox H, Farndon P, Moss C, Grindulis H, Risteli M, Robins SP, Myllyla R (2008) A connective tissue disorder caused by mutations of the lysyl hydroxylase 3 gene. Am J Hum Genet 83 (4):495-503. doi:10.1016/j.ajhg.2008.09.004

24. Micha D, Guo DC, Hilhorst-Hofstee Y, van Kooten F, Atmaja D, Overwater E, Cayami FK, Regalado ES, van Uffelen R, Venselaar H, Faradz SM, Vriend G, Weiss MM, Sistermans EA, Maugeri A, Milewicz DM, Pals G, van Dijk FS (2015) SMAD2 Mutations Are Associated with Arterial Aneurysms and Dissections. Hum Mutat 36 (12):1145-1149. doi:10.1002/humu.22854

25. Thorvaldsdottir H, Robinson JT, Mesirov JP (2013) Integrative Genomics Viewer (IGV): high-performance genomics data visualization and exploration. Brief Bioinform 14 (2):178-192. doi:10.1093/bib/bbs017

26. Robinson JT, Thorvaldsdottir H, Wenger AM, Zehir A, Mesirov JP (2017) Variant Review with the Integrative Genomics Viewer. Cancer Res 77 (21):e31-e34. doi:10.1158/0008-5472.CAN-17-0337

27. Ioannidis NM, Rothstein JH, Pejaver V, Middha S, McDonnell SK, Baheti S, Musolf A, Li Q, Holzinger E, Karyadi D, Cannon-Albright LA, Teerlink CC, Stanford JL, Isaacs WB, Xu J, Cooney KA, Lange EM, Schleutker J, Carpten JD, Powell IJ, Cussenot O, Cancel-Tassin G, Giles GG, MacInnis RJ, Maier C, Hsieh CL, Wiklund F, Catalona WJ, Foulkes WD, Mandal D, Eeles RA, Kote-Jarai Z, Bustamante CD, Schaid DJ, Hastie T, Ostrander EA, Bailey-Wilson JE, Radivojac P, Thibodeau SN, Whittemore AS, Sieh W (2016) REVEL: An Ensemble Method for Predicting the Pathogenicity of Rare Missense Variants. Am J Hum Genet 99 (4):877-885. doi:10.1016/j.ajhg.2016.08.016

28. Jagadeesh KA, Wenger AM, Berger MJ, Guturu H, Stenson PD, Cooper DN, Bernstein JA, Bejerano G (2016) M-CAP eliminates a majority of variants of uncertain significance in clinical exomes at high sensitivity. Nat Genet 48 (12):1581-1586. doi:10.1038/ng.3703

29. Kircher M, Witten DM, Jain P, O'Roak BJ, Cooper GM, Shendure J (2014) A general framework for estimating the relative pathogenicity of human genetic variants. Nat Genet 46 (3):310-315. doi:10.1038/ng.2892

30. Alirezaie N, Kernohan KD, Hartley T, Majewski J, Hocking TD (2018) ClinPred: Prediction Tool to Identify Disease-Relevant Nonsynonymous Single-Nucleotide Variants. Am J Hum Genet 103 (4):474-483. doi:10.1016/j.ajhg.2018.08.005

31. Desmet FO, Hamroun D, Lalande M, Collod-Beroud G, Claustres M, Beroud C (2009) Human Splicing Finder: an online bioinformatics tool to predict splicing signals. Nucleic acids research 37 (9):e67. doi:10.1093/nar/gkp215

32. Hebsgaard SM, Korning PG, Tolstrup N, Engelbrecht J, Rouze P, Brunak S (1996) Splice site prediction in Arabidopsis thaliana pre-mRNA by combining local and global sequence information. Nucleic acids research 24 (17):3439-3452

33. Yeo G, Burge CB (2004) Maximum entropy modeling of short sequence motifs with applications to RNA splicing signals. J Comput Biol 11 (2-3):377-394. doi:10.1089/1066527041410418

34. Reese MG, Eeckman FH, Kulp D, Haussler D (1997) Improved splice site detection in Genie. J Comput Biol 4 (3):311-323. doi:10.1089/cmb.1997.4.311

35. Ashburner M, Ball CA, Blake JA, Botstein D, Butler H, Cherry JM, Davis AP, Dolinski K, Dwight SS, Eppig JT, Harris MA, Hill DP, Issel-Tarver L, Kasarskis A, Lewis S, Matese JC, Richardson JE, Ringwald M, Rubin GM, Sherlock G (2000) Gene ontology: tool for the unification of biology. The Gene Ontology Consortium. Nat Genet 25 (1):25-29. doi:10.1038/75556

36. Verstraeten A, Luyckx I, Loeys B (2017) Aetiology and management of hereditary aortopathy. Nature reviews Cardiology 14 (4):197-208. doi:10.1038/nrcardio.2016.211

37. Stark C, Breitkreutz BJ, Reguly T, Boucher L, Breitkreutz A, Tyers M (2006) BioGRID: a general repository for interaction datasets. Nucleic acids research 34 (Database issue):D535-539. doi:10.1093/nar/gkj109

38. Chatr-Aryamontri A, Oughtred R, Boucher L, Rust J, Chang C, Kolas NK, O'Donnell L, Oster S, Theesfeld C, Sellam A, Stark C, Breitkreutz BJ, Dolinski K, Tyers M (2017) The BioGRID interaction database: 2017 update. Nucleic acids research 45 (D1):D369-D379. doi:10.1093/nar/gkw1102

39. Orchard S, Ammari M, Aranda B, Breuza L, Briganti L, Broackes-Carter F, Campbell NH, Chavali G, Chen C, del-Toro N, Duesbury M, Dumousseau M, Galeota E, Hinz U, Iannuccelli M, Jagannathan S, Jimenez R, Khadake J, Lagreid A, Licata L, Lovering RC, Meldal B, Melidoni AN, Milagros M, Peluso D, Perfetto L, Porras P, Raghunath A, Ricard-Blum S, Roechert B, Stutz A, Tognolli M, van Roey K, Cesareni G, Hermjakob H (2014) The MIntAct project--IntAct as a common curation platform for 11 molecular interaction databases. Nucleic acids research 42 (Database issue):D358-363. doi:10.1093/nar/gkt1115

40. The UniProt C (2017) UniProt: the universal protein knowledgebase. Nucleic acids research 45 (D1):D158-D169. doi:10.1093/nar/gkw1099

41. Finn RD, Coggill P, Eberhardt RY, Eddy SR, Mistry J, Mitchell AL, Potter SC, Punta M, Qureshi M, Sangrador-Vegas A, Salazar GA, Tate J, Bateman A (2016) The Pfam protein families database: towards a more sustainable future. Nucleic acids research 44 (D1):D279-285. doi:10.1093/nar/gkv1344

42. Uhlen M, Fagerberg L, Hallstrom BM, Lindskog C, Oksvold P, Mardinoglu A, Sivertsson A, Kampf C, Sjostedt E, Asplund A, Olsson I, Edlund K, Lundberg E, Navani S, Szigyarto CA, Odeberg J, Djureinovic D, Takanen JO, Hober S, Alm T, Edqvist PH, Berling H, Tegel H, Mulder J, Rockberg J, Nilsson P, Schwenk JM, Hamsten M, von Feilitzen K, Forsberg M, Persson L, Johansson F, Zwahlen M, von Heijne G, Nielsen J, Ponten F (2015) Proteomics. Tissue-based map of the human proteome. Science 347 (6220):1260419. doi:10.1126/science.1260419

43. Grennan AK (2006) Genevestigator. Facilitating web-based gene-expression analysis. Plant Physiol 141 (4):1164-1166. doi:10.1104/pp.104.900198

44. Krawczak M, Nikolaus S, von Eberstein H, Croucher PJ, El Mokhtari NE, Schreiber S (2006) PopGen: population-based recruitment of patients and controls for the analysis of complex genotype-phenotype relationships. Community Genet 9 (1):55-61. doi:10.1159/000090694

45. Lieb W, Jacobs G, Wolf A, Richter G, Gaede KI, Schwarz J, Arnold N, Bohm R, Buyx A, Cascorbi I, Franke A, Glinicke C, Held-Feindt J, Junker R, Kalthoff H, Kramer HH, Leypoldt F, Maass N, Maetzler W, May S, Mehdorn HM, Rocken C, Schafmayer C, Schrappe M, Schreiber S, Sebens S, Stephani U, Synowitz M, Weimer J, Zabel P, Nothlings U, Roder C, Krawczak M (2019) Linking pre-existing biorepositories for medical research: the PopGen 2.0 Network. J Community Genet 10 (4):523-530. doi:10.1007/s12687-019-00417-8

46. Online Mendelian Inheritance in Man, OMIM®. McKusick-Nathans Institute of Genetic Medicine, Johns Hopkins University (Baltimore, MD), November 3, 2017.

47. Coordinators NR (2017) Database Resources of the National Center for Biotechnology Information. Nucleic acids research 45 (D1):D12-D17. doi:10.1093/nar/gkw1071

48. Richards S, Aziz N, Bale S, Bick D, Das S, Gastier-Foster J, Grody WW, Hegde M, Lyon E, Spector E, Voelkerding K, Rehm HL, Committee ALQA (2015) Standards and guidelines for the interpretation of sequence variants: a joint consensus recommendation of the American College of Medical Genetics and Genomics and the Association for Molecular Pathology. Genet Med 17 (5):405-424. doi:10.1038/gim.2015.30

49. Biesecker LG, Harrison SM (2018) The ACMG/AMP reputable source criteria for the interpretation of sequence variants. Genet Med. doi:10.1038/gim.2018.42

50. Hall JG, Flora C, Scott CI, Jr., Pauli RM, Tanaka KI (2004) Majewski osteodysplastic primordial dwarfism type II (MOPD II): natural history and clinical findings. American journal of medical genetics Part A 130A (1):55-72. doi:10.1002/ajmg.a.30203

51. Liu W, Morito D, Takashima S, Mineharu Y, Kobayashi H, Hitomi T, Hashikata H, Matsuura N, Yamazaki S, Toyoda A, Kikuta K, Takagi Y, Harada KH, Fujiyama A, Herzig R, Krischek B, Zou L, Kim JE, Kitakaze M, Miyamoto S, Nagata K, Hashimoto N, Koizumi A (2011) Identification of RNF213 as a susceptibility gene for moyamoya disease and its possible role in vascular development. PLoS One 6 (7):e22542. doi:10.1371/journal.pone.0022542

52. Richards MW, O'Regan L, Mas-Droux C, Blot JM, Cheung J, Hoelder S, Fry AM, Bayliss R (2009) An autoinhibitory tyrosine motif in the cell-cycle-regulated Nek7 kinase is released through binding of Nek9. Mol Cell 36 (4):560-570. doi:10.1016/j.molcel.2009.09.038

53. Moniz L, Dutt P, Haider N, Stambolic V (2011) Nek family of kinases in cell cycle, checkpoint control and cancer. Cell Div 6:18. doi:10.1186/1747-1028-6-18

54. Schurpf T, Chen Q, Liu JH, Wang R, Springer TA, Wang JH (2012) The RGD finger of Del-1 is a unique structural feature critical for integrin binding. FASEB journal : official publication of the Federation of American Societies for Experimental Biology 26 (8):3412-3420. doi:10.1096/fj.11-202036

55. Lee S, Emond MJ, Bamshad MJ, Barnes KC, Rieder MJ, Nickerson DA, Team NGESP-ELP, Christiani DC, Wurfel MM, Lin X (2012) Optimal unified approach for rare-variant association testing with application to small-sample case-control whole-exome sequencing studies. Am J Hum Genet 91 (2):224-237. doi:10.1016/j.ajhg.2012.06.007

56. Lee S, Miropolsky L, Wu M (2017) SKAT: SNP-Set (Sequence) Kernel Association Test. R package version 1.3.2.1. https://CRANR-projectorg/package=SKAT

57. Davies RB (1980) Algorithm AS 155: The Distribution of a Linear Combination of chi-2 Random Variables. Journal of the Royal Statistical Society Series C 29:323-333

58. R Core Team (2019) R: A language and environment for statistical computing. R Foundation for Statistical Computing. URL https://wwwR-projectorg/

59. McCarthy MI, Abecasis GR, Cardon LR, Goldstein DB, Little J, Ioannidis JP, Hirschhorn JN (2008) Genome-wide association studies for complex traits: consensus, uncertainty and challenges. Nat Rev Genet 9 (5):356-369. doi:10.1038/nrg2344

60. Pettersen EF, Goddard TD, Huang CC, Couch GS, Greenblatt DM, Meng EC, Ferrin TE (2004) UCSF Chimera--a visualization system for exploratory research and analysis. J Comput Chem 25 (13):1605-1612. doi:10.1002/jcc.20084

61. Carafoli F, Mayer MC, Shiraishi K, Pecheva MA, Chan LY, Nan R, Leitinger B, Hohenester E (2012) Structure of the discoidin domain receptor 1 extracellular region bound to an inhibitory Fab fragment reveals features important for signaling. Structure 20 (4):688-697. doi:10.1016/j.str.2012.02.011

**SuppLementARY TABLES**

**Table S1**. Selection criteria and constraints of patients for exome and Sanger sequencing.

| **Proband** | **UIA**  **[count]** | **(a)SAH**  **[count]** | **Family history**  **(details)** | **Informed consent** | **Sequencing Technique** | **Remarks** |
| --- | --- | --- | --- | --- | --- | --- |
| **IA6** | 4 | 2 | neg. | ES | ES |  |
| **IA7** | 2 | 0 | pos. (mo, 2 si) | ES | ES | study family |
| **IA8** | 1 | 1 | pos. (mo, 2 si) | ES | ES | study family |
| **IA13** | 2 | 1 | neg. | ES | ES |  |
| **IA15** | 6 | 0 | pos. (mo, 2 si) | ES | ES | study family |
| **IA17** | 1 | 1 | neg. | ES | ES |  |
| **IA18** | 1 | 1 | pos. (1 si) | ES | ES |  |
| **IA24** | 1 | 1 | pos. (2 si, fa, gf) | ES | ES |  |
| **IA40** | 1 | 1 | neg. | ES | ES |  |
| **IA47** | 1 | 1 | pos. (1 si) | ES | ES |  |
| **IA49^a^** | 1 | 0 | pos. (1 si) | ES | ES | AA**^a^** |
| **IA50** | 1 | 2 | pos. (2 da) | ES | ES |  |
| **IA53** | 6 | 1 | neg. | ES | ES |  |
| **IA54** | 5 | 0 | neg. | ES | ES |  |
| **IA57** | 4 | 2 | neg. | ES | ES |  |
| **IA58** | 5 | 1 | neg. | ES | ES |  |
| **IA59^b^** | 1 | 0 | pos. (2 si, gm) | ES | ES | >1 affected relatives**^b^** |
| **IA60** | 3 | 0 | pos. (1 si, mo, ne) | ES | ES |  |
| **IA61** | 4 | 1 | neg. | ES | ES |  |
| **IA63** | 2 | 1 | neg. | ES | ES |  |
| **IA64^b^** | 1 | 0 | pos. (si, mo) | ES | ES | >1 affected relatives**^b^** |
| **IA69** | 3 | 0 | neg. | ES | ES |  |
| **IA70** | 2 | 1 | neg. | ES | ES |  |
| **IA71** | 2 | 1 | neg. | ES | ES |  |
| **IA72** | 1 | 1 | neg. | ES | ES |  |
| **IA73** | 3 | 1 | pos. (1 si) | ES | ES |  |
| **IA74** | 1 | 1 | pos. (n.i.) | ES | ES |  |
| **IA75** | 1 | 1 | neg. | ES | ES |  |
| **IA76** | 4 | 0 | pos. (fa, gm) | ES | ES |  |
| **IA77** | 4 | 0 | neg. | ES | ES |  |
| **IA78^b^** | 1 | 0 | pos. (so, 2 re) | ES | ES | >1 affected relatives**^b^**, giant aneurysma |
| **IA79** | 2 | 0 | pos. (mo) | ES | ES |  |
| **IA83** | 1 | 1 | neg. | ES | ES |  |
| **IA84^b^** | 1 | 0 | pos. (mo, 1 re) | ES | ES | >1 affected relatives**^b^** |
| **IA85** | 4 | 1 | pos. (fa) | ES | ES |  |
| **IA90** | 3 | 0 | pos. (2 si) | ES | ES |  |
| **IA92** | 1 | 1 | pos. (n.i.) | ES | ES |  |
| **IA95** | 3 | 0 | pos. (1 si) | ES | ES |  |
| **SAH39** | 0 | 1 | pos. (1 si) | ES | ES |  |
| **SAH41** | 0 | 1 | pos. (n.i.) | ES | ES |  |
| **SAH45** | 0 | 1 | pos. (1 si) | ES | ES |  |
| **IA1** | 1 | 0 | pos. (1 si) | ES | SS |  |
| **IA3** | 3 | 1 | neg. | SS | SS |  |
| **IA4** | 1 | 1 | neg. | SS | SS |  |
| **IA5** | 2 | 1 | neg. | SS | SS |  |
| **IA9** | 5 | 0 | neg. | SS | SS |  |
| **IA10** | 2 | 0 | neg. | SS | SS | 1 CA, 2 AA**^c^** |
| **IA11** | 2 | 3 | neg. | SS | SS |  |
| **IA12** | 1 | 1 | neg. | SS | SS |  |
| **IA34** | 3 | 1 | neg. | SS | SS |  |
| **IA35** | 1 | 1 | neg. | SS | SS |  |
| **IA36** | 1 | 1 | neg. | SS | SS |  |
| **IA37** | 1 | 1 | neg. | SS | SS |  |
| **IA38** | 1 | 1 | pos. (1 si) | ES | SS |  |
| **IA42** | 1 | 0 | neg. | SS | SS | giant aneurysma |
| **IA46** | 1 | 2 | neg. | SS | SS |  |
| **IA48** | 1 | 1 | neg. | ES | SS |  |
| **IA51** | 2 | 1 | pos. (1 si) | ES | SS |  |
| **IA55** | 1 | 1 | neg. | ES | SS |  |
| **IA56** | 1 | 1 | neg. | ES | SS |  |
| **IA62** | 1 | 0 | neg. | ES | SS | giant aneurysma |
| **IA65** | 1 | 1 | neg. | ES | SS |  |
| **IA66^c^** | 0 | 0 | pos. (1 si, so, un) | ES | SS | TAA and >1 affected relatives**^d^** |
| **IA81** | 1 | 1 | neg. | ES | SS |  |
| **IA82** | 1 | 0 | neg. | ES | SS |  |
| **IA86** | 1 | 1 | neg. | ES | SS |  |
| **IA87** | 1 | 1 | neg. | ES | SS |  |
| **IA88** | 1 | 0 | pos. (2 si) | ES | SS |  |
| **IA96** | 1 | 1 | pos. (1 si) | ES | SS |  |
| **IA97** | 1 | 1 | neg. | ES | SS |  |
| **IA98** | 1 | 1 | neg. | ES | SS |  |
| **IA99** | 1 | 0 | pos. (fa) | ES | SS | giant aneurysma |
| **IA102** | multiple | 0 | neg. | ES | SS |  |
| **IA104** | 1 | 1 | neg. | ES | SS |  |
| **IA105** | 1 | 0 | neg. | ES | SS |  |
| **IA106** | 2 | 1 | neg. | ES | SS |  |
| **IA107** | 1 | 1 | neg. | ES | SS |  |
| **IA108** | 1 | 0 | pos. (n.i.) | ES | SS |  |

**Table S1**. Selection criteria and constraints of probands for exome and Sanger sequencing. Patient ID, number of unruptured intracranial aneurysms (UIA) and (aneurysmal) subarachnoid hemorrhage [(a)SAH], details on family history as well as type of available informed consent is given for each individual. If available, details on family history are indicated as degrees of relationship and numbers of affected relatives. 38 patients with aneurysmal SAH and/or UIA including three family members (indicated by “study family”) were exome sequenced (ES); 37 patients were Sanger sequenced (SS). One sporadic patient and two siblings with SAH but without angiographic aneurysm evidence were also exome sequenced; these patients are indicated by “SAH”. Neg., negative family history; pos., positive family history; ES exome sequencing; SS, Sanger sequencing; n.i., no information; si, sibling; mo, mother; fa, father; so, son; da, daughter; gm, grandmother; gf, grandfather; ne, nephew; un, uncle; re, unspecified relatives.

**^a^**, IA49 has an aortic aneurysm (AA) in addition to IA, suggesting a generalized vascular disorder.

**^b^**, IA59, IA64, IA78 and IA84 have more than one affected relatives with UIA/aSAH; thus a genetic background is likely.

**^c^**, IA10 had a carotid aneurysm (CA) and 2 aortic aneurysms (AA) in addition to 2 IAs.

**^d^**, IA66 has a thoracic aortic aneurysm (TAA); his father, sister and son died due to SAH.

**Table S2** Known disease genes for vascular/connective tissue disorders

| **Gene** | **Phenotype** | ***MIM* number** or **reference** |
| --- | --- | --- |
| *ACTA2* | familial thoracic aortic aneurysm 6 | #611788 |
| *ADAMTS2* | Ehlers-Danlos syndrome type VIIC | #225410 |
| *B3GALT6* | Ehlers-Danlos syndrome progeroid type 2 | #615349 |
| *B4GALT7* | Ehlers-Danlos syndrome with short stature and limb anomalies | #130070 |
| *BGN* | Meester-Loeys syndrome | #300989 |
| *CHST14* | Ehlers-Danlos syndrome musculocontractural type 1 | #601776 |
| *COL1A1* | Ehlers-Danlos syndrome classic type | #130000 |
|  | Ehlers-Danlos syndrome type VIIA | #130060 |
| *COL1A2* | Ehlers-Danlos syndrome type VIIB | #130060 |
| *COL3A*1 | Ehlers-Danlos syndrome type IV | #130050 |
| *COL5A1* | Ehlers-Danlos syndrome classic type | #130000 |
| *COL5A2* | Ehlers-Danlos syndrome classic type | #130000 |
| *DCHS1* | Mitral valve prolapse 2 | #607829 |
| *EFEMP2* | Cutis laxa autosomal recessive type IB | #614437 |
| *ELN* | Cutis laxa autosomal dominant | #123700 |
| *EMILIN1* | n.r. | Capuano et al. (2016) [19] |
| *FBLN5* | Cutis laxa autosomal dominant 2 | #614434 |
| *FBN1* | Marfan syndrome | #154700 |
| *FBN2* | Contractural arachnodactyly congenital | #121050 |
| *FKBP14* | Ehlers-Danlos syndrome with progressive kyphoscoliosis, myopathy and hearing loss | #614557 |
| *FLNA* | Cardiac valvular dysplasia X-linked | #314400 |
| *FOXE3* | Aortic aneurysm, familial thoracic 11, susceptibility to | #617349  Kuang et al. (2016) [20] |
| *GANAB* | Polycystic kidney disease 3 | #600666 |
| *LOX* | Aortic aneurysm, familial thoracic 10 | #617168  Lee et al. (2016) [21] |
| *LTBP2* | Weill-Marchesani syndrome 3 recessive | #614819 |
| *LTBP4* | Cutis laxa autosomal recessive type IC | #613177 |
| *MAT2A* | n.r. | Guo et al. (2015) [22] |
| *MFAP5* | Aortic aneurysm familial thoracic 9 | #616166 |
| *MYH11* | Aortic aneurysm familial thoracic 4 | #132900 |
| *MYLK* | Aortic aneurysm familial thoracic 7 | #613780 |
| *NOTCH1* | Aortic valve disease 1 | #109730 |
| *PKD1* | Polycystic kidney disease 1 | #173900 |
| *PKD2* | Polycystic kidney disease 2 | #613095 |
| *PLOD1* | Ehlers-Danlos syndrome type VI | #225400 |
| *PLOD3* | Lysyl hydroxylase 3 deficiency | #612394  Salo et al. (2008) [23] |
| *PRDM5* | Brittle cornea syndrome 2 | #614170 |
| *PRKG1* | Aortic aneurysm familial thoracic 8 | #615436 |
| *SKI* | Shprintzen-Goldberg syndrome | #182212 |
| *SLC2A10* | Arterial tortuosity syndrome | #208050 |
| *SLC39A13* | Spondylocheirodysplasia/Ehlers-Danlos syndrome-like | #612350 |
| *SMAD2* | n.r. | Micha et al. (2015) [24] |
| *SMAD3* | Loeys-Dietz syndrome 3 | #613795 |
| *SMAD4* | Myhre syndrome | #139210 |
| *TGFB2* | Loeys-Dietz syndrome 4 | #614816 |
| *TGFB3* | Loeys-Dietz syndrome 5 | #615582 |
| *TGFBR1* | Loeys-Dietz syndrome 1 | #609192 |
| *TGFBR2* | Loeys-Dietz syndrome 2 | #610168 |
| *TNXB* | Ehlers-Danlos syndrome due to tenascin X deficiency | #606408 |
| *ZNF469* | Brittle cornea syndrome 1 | #229200 |

**Table S2** Known disease genes for vascular/connective tissue disorders. *MIM*, Mendelian Inheritance in Man; n.r., none reported.

**Table S3** Functional relevance of genes with top ranked putative pathogenic variants shared by three affected siblings.

| Gene | Transcript ID | c. notation  p. notation | Functional relevance |
| --- | --- | --- | --- |
| *NEK4* | NM_003157 | c.190A>T  p.Asn64Tyr | I+ A+ |
| *EDIL3* | NM_005711 | c.383G>A  p.Cys128Tyr | O+ I+ A+ E+ |
| *EDNRB* | NM_001201397 | c.891T>G  p.Ser297Arg | O+ A+ E+ |
| *DNAH9* | NM_001372 | c.13304T>C  p.Ile4435Thr |  |
| *GGA3* | NM_138619 | c.16G>A  p.Gly6Arg | A+ |

**Table S3.** Functional relevance of genes with top ranked putative pathogenic variants shared by three affected siblings. Transcript IDs correspond to the NCBI Reference Sequence (RefSeq) project. Nucleotide and amino acid changes are given according to the indicated transcript ID. Nucleotide numbering uses +1 as the A of the ATG translation initiation codon in the reference sequence, with the initiation codon as codon 1. Variants were ranked according to their functional relevance regarding vascular biology by using various databases and repositories; functional relevance was determined as described in the **Supplementary Methods**: O+ = gene ontology positive; I+ = interaction positive; A+ = amino acid positive; E+ = expression positive.

**Table S4** Variants in top ranked disease candidate genes identified in exomes from 35 unrelated individuals and 3 relatives.

| Gene  Transcript ID | c.:p. notation | dbSNP^a^ Accession Number | gnomAD^b^  MAF | PopGen/ IKMB-controls^c^  MAF | Pathogenicity predictions^d^  CADD/REVEL/M-CAP/ClinPred | Patient ID |
| --- | --- | --- | --- | --- | --- | --- |
| *NEK4*  NM_003157 | c.190A>T:p.Asn64Tyr | n.l. | 0 | 0 | **23.7**/0.452/**0.113**/**0.984** | IA7, IA8, IA15 |
|  | c.1699T>C:p.Phe567Leu | rs34986855 | 0.02391 | 0 | 0.12/0.064/n.a./0.001 | IA18,IA71,IA76,IA78,IA24 |
| *EDIL3*  NM_005711 | c.383G>A:p.Cys128Tyr | n.l. | 0 | 0 | **29.1**/**0.946**/**0.523**/**0.998** | IA7, IA8, IA15 |
|  | c.1159A>C:p.Lys387Gln | rs552874498 | 0.000131 | 0.000101 | **24.5**/**0.723**/**0.266**/0.078 | IA13 |
| *EDNRB*  NM_001201397 | c.891T>G:p.Ser297Arg | n.l. | 0 | 0 | **23.7**/0.303/0.02/**0.972** | IA7, IA8, IA15 |
|  | c.1048G>T:p.Val350Phe | rs77132068 | 0.0008505 | 0.002301 | 6.7/0.057/0.021/0.002 | IA63 |
|  | c.56T>A:p.Leu19Gln | rs201560121 | 0.0000184 | 0.000101 | 13.7/0.117/0.033/**0.763** | IA17 |
| *DNAH9*  NM_001372 | c.13304T>C:p.Ile4435Thr | n.l. | 0 | 0 | **24.5**/0.203/0.01/**0.983** | IA7, IA8, IA15 |
|  | c.41_42ins25:p.Asn14fs | rs761517350 | 0.0237 | 0 | n.a./n.a./n.a./n.a. | IA70 |
|  | c.1571G>A:p.Arg524Gln | rs140374295 | 0.0001628 | 0.001087 | 16.1/0.282/0.016/0.103 | IA58,IA85 |
|  | c.1810A>G:p.Met604Val | rs61740059 | 0.02278 | 0.03381 | **24.2**/0.381/n.a./0.026 | IA49,IA74,IA76 |
| *GGA3*  NM_138619 | c.16G>A:p.Gly6Arg | n.l. | 0 | 0 | **33.0**/0.299/**0.049**/**0.988** | IA7, IA8, IA15 |
|  | c.1769C>T:p.Ser590Leu | rs146451191 | 0.001355 | 0.002327 | **32.0**/0.313/0.018/0.082 | IA78 |
|  | c.656A>G:p.Glu219Gly | rs52809447 | 0.007505 | 0.011076 | **30.0**/ 0.397/n.a./0.035 | IA75, SAH45 |

**Table S4.** Variants in top ranked disease candidate genes. Sequence alterations were identified by exploring available exome sequencing data as described in the text. Nucleotide and amino acid changes are given according to the indicated transcript ID. Nucleotide numbering uses +1 as the A of the ATG translation initiation codon in the reference sequence, with the initiation codon as codon 1. n.l. not listed; n.a., not applicable.

^a^, dbSNP: The Single Nucleotide Polymorphism Database (dbSNP) of Nucleotide Sequence Variation (https://www.ncbi.nlm.nih.gov/snp).

^b^, gnomAD, Genome Aggregation Database (v2.1.1) [16]; all MAF, minor allele frequency (total population).

^c^, PopGen/IKMB-controls, PopGen database for population-based and disease-specific issues to study molecular and non-molecular risk factors for numerous disorders [44,45]/healthy control samples collected in the Institute of Clinical Molecular Biology in Kiel; MAF minor allele frequency.

^d^, Details on pathogenicity predictors and respective thresholds [CADD (≥20), REVEL (≥0.5), M-CAP (≥0.025), ClinPred (≥0.5)] are given in the **Supplementary Methods**. Scores ≥ thresholds are shown in bold.

**Table S5** Association tests on unknown and very rare coding variants (MAF ≤ 0.001) in *EDIL3*.

| **Statistical test** | **Study cohort** | **Controls** | **p-value** |
| --- | --- | --- | --- |
| SKAT | 3/73 [carrier] | 0/100 [carrier]  Hamburg controls | 0.0298 |
| Burden test |  |  | 0.0152 |
| SKAT | 3/73 [carrier] | 326/64,603  [allele count]  gnomAD | 2.53e-33 |
| Burden test |  |  | 0.0000150 |
| Fisher’s exact test | 3/73 [carrier] | 326/64,603  [allele count]  gnomAD | 0.00624  (OR=8.45; 95% CI 1.69 – 26.0) |

**Table S6** Variant in *RNF213* identified in 2 siblings with SAH.

| c. notation  p. notation | dbSNP rs ID  ClinVar allele ID | Classifi-  cation^a^ | gnomAD all MAF^b^  AC/NoH/AN^b^ | Pathogenicity predictions^c^ | |
| --- | --- | --- | --- | --- | --- |
|  |  |  |  | **CADD/REVEL/**  **M-CAP/ClinPred** | **Impact on splicing** |
| c.10450G>A  p.Gly3484Ser | rs140300282  n.r. | VUS_PP2+BP4_ | 0.0004128  115/0/278596 | 7.512/0.019/  0.026/0.018 | n.d. |

**Table S6** Variant in *RNF213* identified in 2 siblings with SAH (**Figure S1B**). Nucleotide and amino acid changes are given according to the *RNF213* transcript ID NM_001256071. Nucleotide numbering uses +1 as the A of the ATG translation initiation codon in the reference sequence, with the initiation codon as codon 1. n.r., not reported; n.d. not determined.

**^a^**, Variant classification: VUS, variant of uncertain significance. Classification criteria PP2 and BP4 are explained in the **Supplementary Methods**.

**^b^**, gnomAD, Genome Aggregation Database (v2.1); all MAF, minor allele frequency (total population); AC/NoH/AN, allele count/number of homozygotes/allele number (i.e. total number of analyzed alleles).

**^c^**, Details on pathogenicity predictors and respective thresholds [CADD (≥20), REVEL (≥0.5), M-CAP (≥0.025), ClinPred (≥0.5)] as well as on splice site predictions with HSF, NetGene2 (NG2), MaxEntScan (MES) and BDGP are given in the **Supplementary Methods**.
