# Supplementary figures and images for: Exome sequencing in 38 patients with intracranial aneurysms and subarachnoid hemorrhage
Source: J Neurol. 2020 May 4;267(9):2533–45. doi: 10.1007/s00415-020-09865-6 (PMC7419486; doi:10.1007/s00415-020-09865-6)

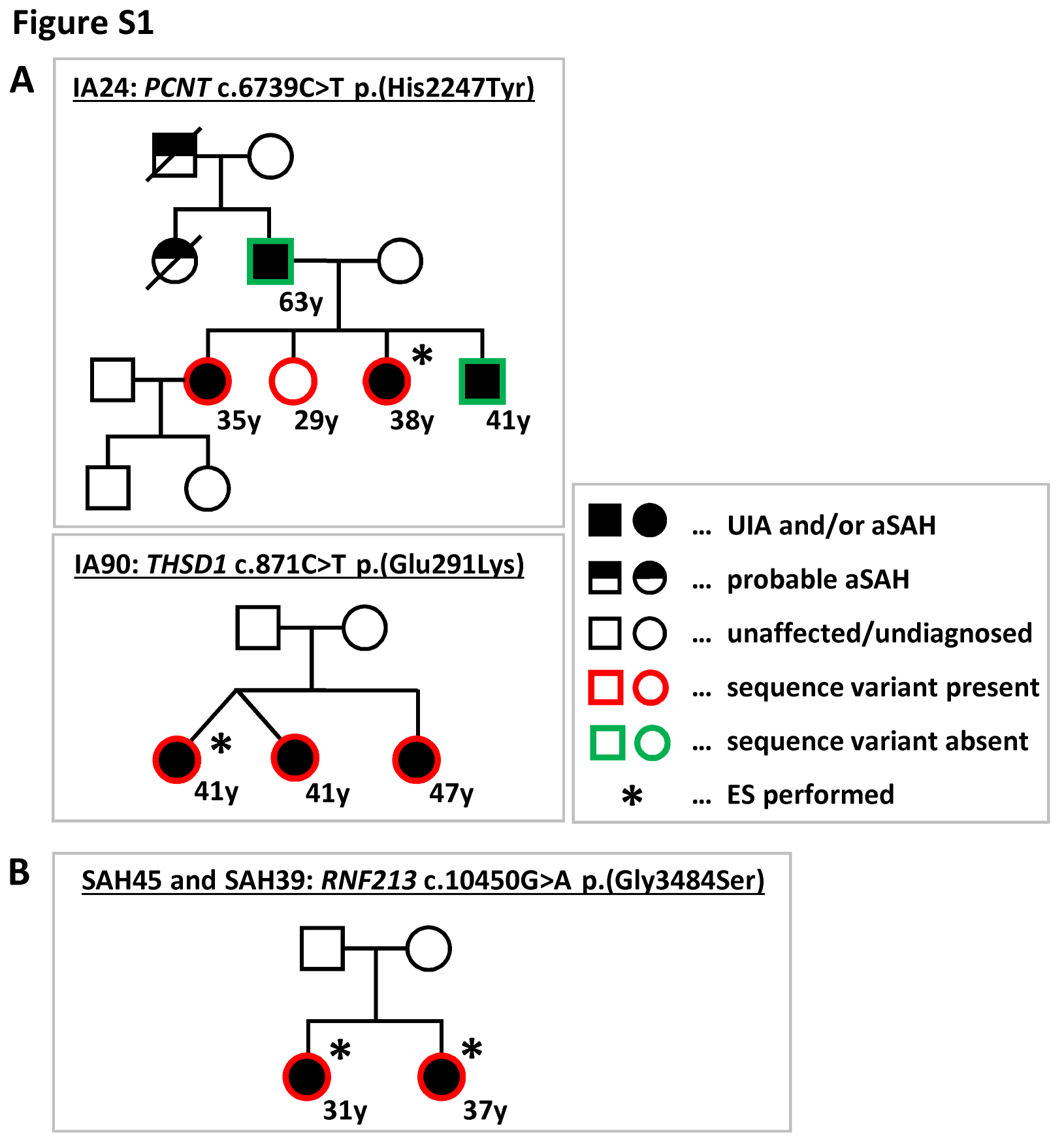

Supplement: Supplementary file 2 — Figure S1. Pedigrees for families of subjects IA24 and IA90 as well as SAH45 and SAH39. Exome sequenced individuals are marked with an asterisk. Criteria for defining the disease phenotypes (UIA and/or aSAH, probable aSAH) are outlined in the methods section. Age at inclusion is given in years (y). Individuals positive (red label) and negative (green label) for the respective sequence variant are indicated. (TIF 195 kb) [file 415_2020_9865_MOESM2_ESM.tif]

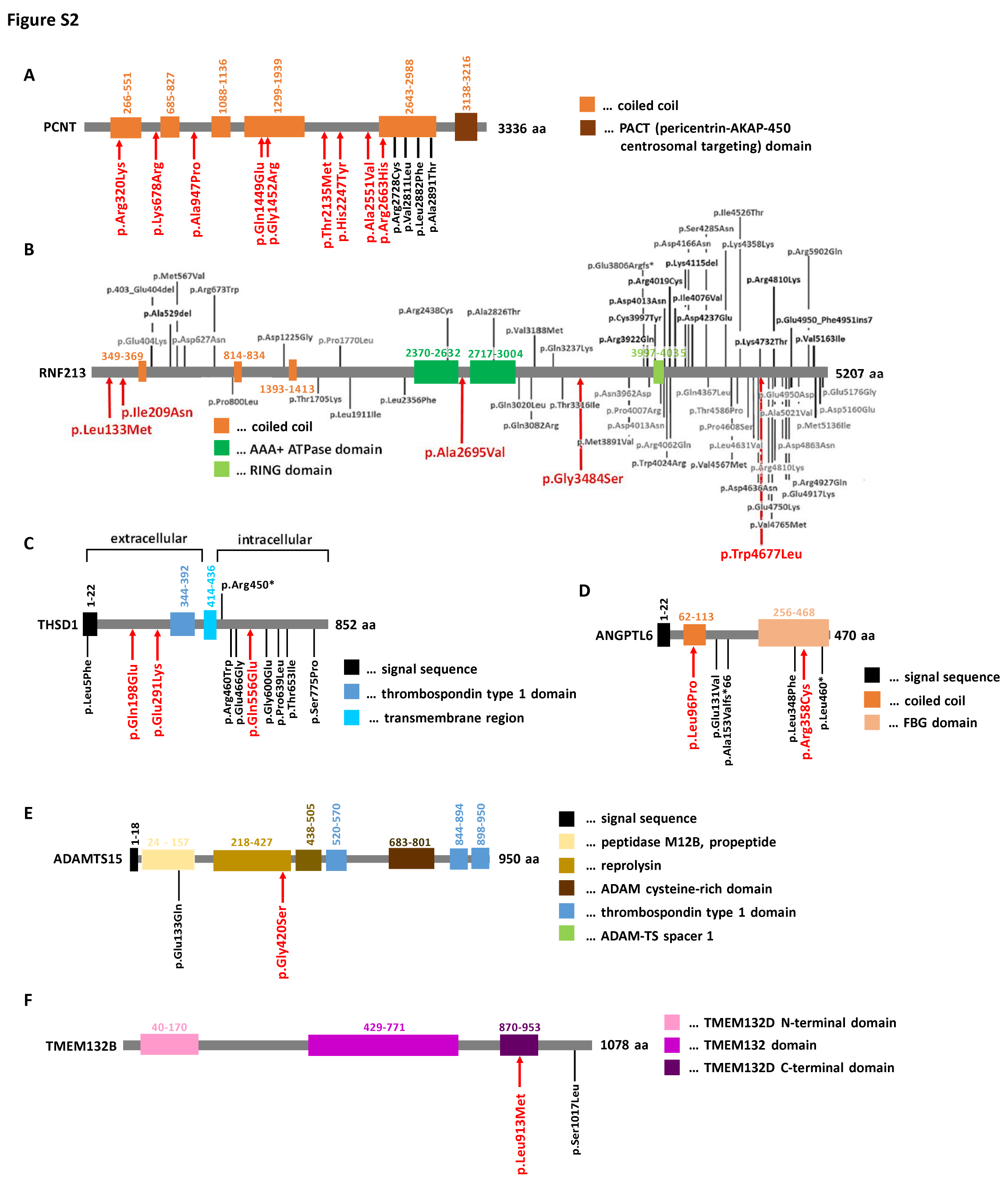

Supplement: Supplementary file 3 — Figure S2. Schematic representation of proteins previously associated with IA/SAH. Distribution of putative disease-associated amino acid changes and protein domains/motifs with position specifications are shown. Figure were designed with the Pfam database. aa, amino acids. A. PCNT variants and protein domains and motifs. Variants identified in this study (red letters) and variants reported in Lorenzo-Betancor et al. (2018) (black letters) are given. B. RNF213 functional variants found in individuals with IA and Moyamoya disease (black and grey letters, respectively); figure was adapted from Zhou et al. (2016). Five variants found in the current study are marked in red. C. THSD1 variants and protein domains and motifs. Variants identified in this study (red letters) and variants reported in Santiago-Sim et al. (2016) (black letters) are given. D. ANGPTL6 variants and protein domains and motifs. Variants identified in this study (red letters) and variants reported in Bourcier et al. (2018) (black letters) are given. E. ADAMTS15 variants and protein domains and motifs. Variants identified in this study (red letters) and variants reported in Yan et al. (2015) (black letters) are given. F. TMEM132B variants and protein domains and motifs. Variants identified in this study (red letters) and variants reported in Farlow et al. (2015) (black letters) are given. (TIF 864 kb) [file 415_2020_9865_MOESM3_ESM.tif]

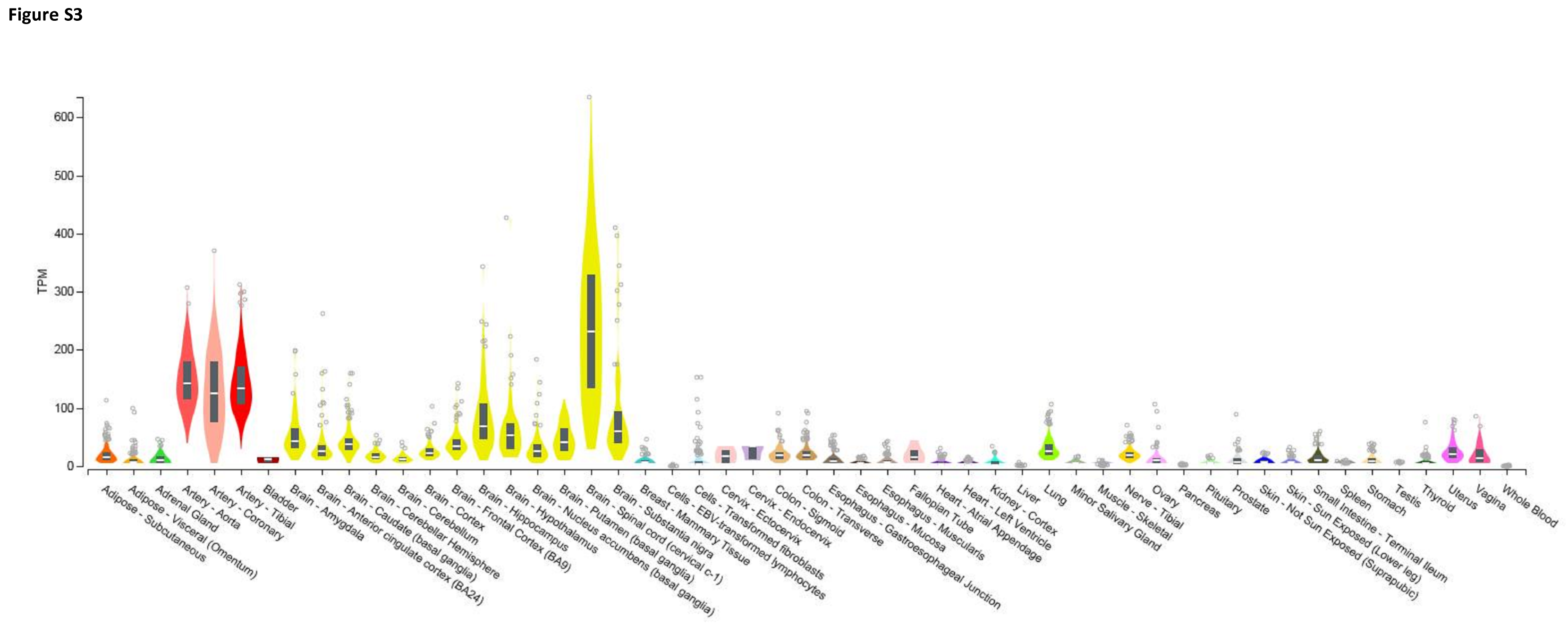

Supplement: Supplementary file 4 — Figure S3. Tissue-specific gene expression of EDIL3. This figure was obtained from the Genotype-Tissue Expression (GTEx) Portal on 02/19/2019 and dbGaP accession number phs000424.v7.p2. The GTEx Project was supported by the Common Fund of the Office of the Director of the National Institutes of Health, and by NCI, NHGRI, NHLBI, NIDA, NIMH, and NINDS. (TIF 1536 kb) [file 415_2020_9865_MOESM4_ESM.tif]
